# Exploring Imaging Applications of a Red-Emitting π-Acceptor (π-A) Pyrene-Benzothiazolium Dye

**DOI:** 10.3390/bios14120612

**Published:** 2024-12-13

**Authors:** Chathura S. Abeywickrama, Enya Huang, Wenhui Yan, Michael A. Vrionides, Paaramitha Warushavithana, Kristen A. Johnson, Robert V. Stahelin, Yi Pang, Tomoyasu Mani, Kaveesha J. Wijesinghe

**Affiliations:** 1 Department of Pharmaceutical Sciences, University of Connecticut, Storrs, CT 06269, USA; 2 Department of Chemistry, University of Connecticut, Storrs, CT 06269, USA; 3 Department of Chemistry, University of Colombo, Colombo 00300, Sri Lanka; 4 Department of Chemistry and Biochemistry, University of Notre Dame, Notre Dame, IN 46556, USA; 5 Borch Department of Medicinal Chemistry and Molecular Pharmacology, Purdue University, West Lafayette, IN 47907, USA; 6 Department of Chemistry, The University of Akron, Akron, OH 44325, USA

**Keywords:** pyrene–benzothiazolium, fluorescence microscopy, cell imaging, fluorescence lifetime, lysosome, Stokes’ shift

## Abstract

Bright biocompatible fluorescent imaging dyes with red to near-infrared (NIR) emissions are ideal candidates for fluorescence microscopy applications. Pyrene–benzothiazolium hemicyanine dyes are a new class of lysosome-specific probes reported on recently. In this work, we conduct a detailed implementation study for a pyrene–benzothiazolium derivative, BTP, to explore its potential imaging applications in fluorescence microscopy. The optical properties of BTP are studied in intracellular environments through advanced fluorescence microscopy techniques, with BTP exhibiting a noticeable shift toward blue (λ_em_ ≈ 590 nm) emissions in cellular lysosomes. The averaged photon arrival time (AAT)-based studies exhibit two different emissive populations of photons, indicating the probe’s dynamic equilibrium between two distinctively different lysosomal microenvironments. Here, BTP is successfully utilized for time-lapse fluorescence microscopy imaging in real-time as a ‘wash-free’ imaging dye with no observed background interference. BTP exhibits an excellent ability to highlight microorganisms (i.e., bacteria) such as *Bacillus megaterium* through fluorescence microscopy. BTP is found to be a promising candidate for two-photon fluorescence microscopy imaging. The two-photon excitability of BTP in COS-7 cells is studied, with the probe exhibiting an excitation maximum at λ_TP_ ≈ 905 nm.

## 1. Introduction

**Scheme 1:**
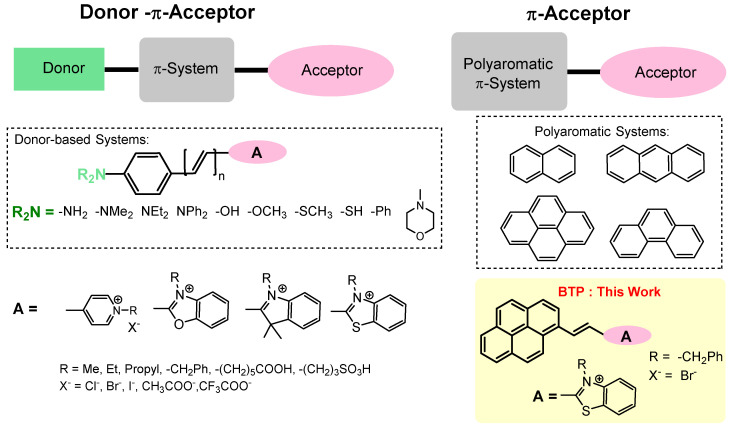
The architecture of donor–π–acceptor and π-acceptor type probes and the chemical structure of BTP.

Small-molecule fluorescent imaging dyes have become a powerful tool to visualize complex biological environments [1,2,3,4,5,6]. Due to their compact size, structural simplicity, and synthetic tunability, the application of small-molecule organic dyes as fluorescence imaging agents has become a popular technique. Fluorophore engineering has become a high-demand research area in biomedical sciences owing to the rapid growth in advanced imaging techniques (i.e., super-resolution microscopy) over the past decades [7,8,9]. An ideal fluorescent imaging dye must produce a stable fluorescence signal while causing minimal perturbations to the microenvironment of the biological specimen. To maintain a stable fluorescence signal, it is important to minimize undesirable self-quenching effects that arise due to the narrow Stokes’ shift of an imaging dye. The excited-state intramolecular proton transfer (ESIPT) and intramolecular charge transfer (ICT) are two well-known Stokes’ shift elongation strategies that mitigate characteristic self-quenching effects in the narrow Stokes’ shift fluorescent probes [2,10,11,12,13,14,15,16]. In the presence of a strong electron acceptor group, certain conjugated molecules can generate an enhanced Stokes’ shift (Δλ > 50 nm) by delocalizing electron density via push and pull ICT mechanisms [15,16,17,18,19,20]. The donor–π–acceptor (D–π–A) and π-acceptor (π-A) are two important classes of ICT-based probes that have been developed to achieve enhanced Stokes’ shifts (Scheme 1) [16,19,20,21,22,23,24]. The observed enhancement in Stokes’ shift and the environmental sensitivity of the probe is dependent on the nature of the electron donor, π-extender, and the electron acceptor in the D–π–A system [17,23,25,26]. The π-acceptor systems are an interesting class of molecules that have been recently reported to exhibit large Stokes’ shifts (Δλ > 100 nm). In this class of probes, a polyaromatic π-system (i.e., pyrene) is served as an electron donor/delocalizer while stabilizing the charge-transfer excited state via resonance stabilization [2,22,27,28]. As a result, a noticeable bathochromic shift in the emission spectra is observed in the π-acceptor systems that leads to a large Stokes’ shift [2,22].

Pyrene is a polyaromatic π system that has been widely used as a structural component in several interesting small-molecule fluorescent imaging probes [29,30,31]. By incorporating a suitable acceptor (A) group into the π system, several interesting small-molecule probes with useful fluorescence microscopy imaging applications have been developed, recently [21,22,27]. Unlike conventional pyrene-centered blue–green emitting dyes, these novel pyrene–hemicyanine imaging probes have unique advantages, such as red to far-red emission, large Stokes’ shift, wash-free staining ability, high fluorescence quantum yield, and good biocompatibility [21,22,27]. Therefore, the pyrene-based π-acceptor probe design has been reported to exhibit a superior photophysical performance [15,17,18,21,27,30]. In our previous work, we have reported on a series of pyrene–benzothiazolium-based π-acceptor probes for fluorescence microscopy imaging of cellular lysosomes [13,21]. These probes exhibit exceptional biocompatibility (20 µM > LC50), wash-free staining ability, and excellent stability, enabling live-cell imaging for long-term periods (i.e., up to 24 h) to study lysosomes [13]. Unlike conventional amine-based lysosome-specific dyes that target acidic lumen (i.e., commercial LysoTracker^TM^ dyes), the new class of pyrene–benzothiazolium probes do not contain any basic functional groups (i.e., amines) that cause significant toxic effects by lysosomal pH elevation (i.e., alkalinizing effect) [21]. Therefore, an in-depth investigation into the photophysical and imaging applications of pyrene–benzothiazolium derivatives is important to discover their full potential as fluorescent imaging probes for visualizing biological systems. In this research article, we aim to expand the scope of understanding of pyrene–benzothiazolium derivative BTP (see Scheme 1) as a fluorescence microscopy imaging agent, while performing a detailed study of its cell imaging applications and photophysical characteristics in biological environments. This work further summarizes the photophysical performance (in solution and cellular environments), multicolor cell imaging ability, time-lapse imaging applications, multiphoton excitation ability (i.e., two-photon fluorescence microscopy), and eukaryotic cell imaging ability of BTP.

## 2. Materials and Methods

All chemicals for spectroscopic analyses, cell culture, and bioimaging experiments were purchased from Sigma-Aldrich (St. Louis, MO, USA), ThermoFisher Scientific (Agawam, MA, USA), and VWR (Radnor, PA, USA) were used as they were received. Other specialized cell culture and fluorescent confocal microscopy reagents and consumables were purchased from Addgene (Watertown, MA, USA), Biotium (Fremont, CA, USA), ThermoFisher (Agawam, MA, USA), and Fisher Scientific (Pittsburgh, PA, USA). All spectroscopic analysis experiments were conducted in spectroscopic grade solvents (ThermoFisher). UV–visible spectroscopy studies were performed in an Agilent Cary 3500 (Santa Clara, CA, USA) UV–visible spectrometer at 25 °C. Fluorescence spectroscopy data were acquired using a FLS 1000 photoluminescence spectrometer (Edinburgh Instruments, Livingston, UK)) and a HORIBA Fluoromax-4 spectrofluorometer (Horiba Instruments, Irvine, CA, USA) at 25 °C. Fluorescence confocal microscopy (single-photon) imaging studies were conducted using a Leica Stellaris 5 white light laser (WLL) (Mannheim, Germany) confocal microscope and a Zeiss LSM 710 confocal microscope (White Plains, NY, USA). Two-photon fluorescence microscopy images were acquired through an Olympus FV1000 multiphoton microscope (Tokyo, Japan). Bacterial cell images were acquired through an Olympus BX53 upright fluorescence microscope (Tokyo, Japan). The synthesis of the probe BTP was conducted based on previously published literature [21] verbatim and no changes were made to the reported methodology. Human bone osteosarcoma (MG-63), human brain glioblastoma (A-172), and *Cercopithecus aethiops* (African green Monkey) kidney cells (COS-7) were used for live-cell imaging experiments. *Bacillus megaterium* bacterial cultures were used for bacteria cell imaging experiments. All fluorescence microscopy images were analyzed using the Fiji ImageJ (1.54f) and LasX (Leica Microsystems) image processing applications. All spectroscopy data were processed through the Origin^®^ 2019 software.

### 2.1. Optical Properties Evaluation

Stock solutions of the BTP were prepared in molecular biology grade DMSO in either 10 mM or 1 mM concentrations and aliquoted and stored at −20 °C. For all steady-state measurements (absorbance and emission), the working concentration of the probe was 1 × 10^−5^ M unless otherwise specified. While acquiring the emission spectra in solution (single-photon excitation), BTP was excited at 490 nm and the emissions were collected from 510 nm to 800 nm (unless otherwise specified) at 25 °C. Two-photon excitations were performed in a dichloromethane and ethanol solution of BTP (1 × 10^−5^ M) while exciting the probe from 900 nm to 1180 nm at 25 °C. The relative fluorescence quantum yields (ϕ_fl_) were calculated using rhodamine 6G as the standard (in ethanol) at 490 nm, while considering that the fluorescence quantum yield of rhodamine 6G is 0.95 in ethanol. The following equation was used for fluorescence quantum yield determination [32,33].

(ϕ_fl_) _sample =_ ϕ_ref_ × (A_ref_/A_sample_) × [(I _sample_)/(I_ref_)] × (ɳ_sample_)^2^/(ɳ_ref_)^2^

where A is the absorbance of the sample, I is the integrated fluorescence intensity, and ɳ is the refractive index of the solvent.

### 2.2. Cell Culture and Staining

MG-63, A-172, and COS-7 cells were grown in Dulbecco’s modified Eagle’s medium (DMEM) (Gibco) containing 10% fetal bovine serum (FBS) and 1% penicillin streptomycin (Penstrep) at 37 °C in a 5% CO_2_ humidity-controlled incubator. Cells were seeded in Nunc^TM^ Lab-Tek^TM^II chambered (8-well) cover glass (#1.5) plates or in 40 mm Nunc^TM^ glass bottom (#1.5) dishes with a 10 mm bottom diameter. Cells were seeded at a density of 50,000–70,000 cells per well and incubated for 24 h prior to staining. The BTP stock solution was made in DMSO at a 1 mM concentration, and the final concentration of the BTP was 1 µM in the stained cells. For staining experiments only with BTP, no post-staining washing step was performed. For co-staining experiments, Hoechst 33342 (1 mg/mL in DPBS), LysoTracker^TM^ green DND-26 (1 mM in DMSO), and MitoView^TM^ Green (0.2 mM in DMSO) dyes were used. The final staining concentrations of the commercial probes were 1 µg/mL (Hoechst 33342), 70 nm (LysoTracker^TM^ green DND-26), and 200 nM (MitoView^TM^ Green), respectively. Only for BTP staining experiments, cells were treated at a 1 µM concentration in FluoroBrite^TM^ DMEM cell imaging solution for 30 min at 37 °C in a 5% CO_2_ humidity-controlled incubator. For co-staining experiments, stained cells were incubated for 30–45 min at 37 °C in a 5% CO_2_ humidity-controlled incubator. For co-staining experiments, a post-staining washing step was performed twice with 1 × PBS prior to imaging. The final DMSO percentage during the staining was maintained below 0.25% (*v/v*) for all imaging experiments. For CellLight^TM^ Lysosomes-GFP expression experiments in COS-7 cells, the recommended manufacturer’s protocol was followed.

### 2.3. Live-Cell Fluorescence Confocal Microscopy

CellLight^TM^ Lysosomes-GFP expressed COS-7 cells co-stained with BTP (1 µM) were imaged using a Zeiss LSM 710 fluorescence confocal microscope with an oil 63 × 1.4 numerical aperture objective. The lysosome GFP was excited with a 488 nm laser line and the emissions were collected from 495 nm to 520 nm. BTP was excited with a 514 nm laser and the emissions were collected from 550 nm to 650 nm. All other co-staining experiments were analyzed using a Leica Stellaris 5 white light laser (WLL) confocal microscope equipped with HyD S detectors. Co-staining experiments were performed under the analogue (intensity mode) setting and frame acquisition settings, with the following acquisition parameters: BTP (λ_ex_ = 520 nm; λ_em_ = 550–700 nm); Hoechst 33342 (λ_ex_ = 405 nm; λ_em_ = 440–470 nm); LysoTracker^TM^ green DND-26 (λ_ex_ = 495 nm; λ_em_ = 505–530 nm); MitoView^TM^ green (λ_ex_ = 490 nm; λ_em_ = 500–540 nm). The LasX dye assistant platform was used to mitigate the cross-talking/noise during the co-staining experiments.

### 2.4. Quantitative Fluorescence Confocal Microscopy

The determination of the excitation (λ_ex_) and emission (λ_em_) maxima of BTP in cellular environments was achieved by exciting stained cell samples with a consistent laser beam with a constant power (0.4 mW) and gain (40%), followed by photon counting detectors. For the excitation analysis, the stained cell samples were excited from 485 nm to 565 nm at 5 nm intervals. For each excitation wavelength, the emission was collected from 570 nm to 720 nm. The resulting fluorescence microscopy images were quantified using the Fiji ImageJ software to calculate the averaged fluorescence intensity for each excitation wavelength. The emission maximum (λ_em_) determination was performed by exciting stained cells at 515 nm (0.4 mW and 40% gain) while collecting emissions from 530 nm to 720 nm at 10 nm intervals. The resulting fluorescence microscopy images were quantified using the Fiji ImageJ software to calculate the averaged fluorescence intensity for each emission window.

Quantitative fluorescence microscopy images (photon counting mode) were acquired for BTP stained cells (1 µM) at a 515 nm excitation (0.4 mW and 40% gain) for the 550 nm to 700 nm emission window. The photon distribution (emitted) based on their averaged arrival time at the detector was recorded using the Las X TauSeperation tool (Leica) [34,35]. Similarly, the average photon distribution was recorded for cells stained with LysoTracker^TM^ red DND-99 (0.1 µM) by exciting them at 560 nm (0.4 mW and 40% gain) and collecting emissions between 575 nm and 700 nm. The Las X TauSeperation tool was used to distinguish the changes in population distributions of the emission photons of BTP and LysoTracker^TM^ red DND-99.

### 2.5. Two-Photon Fluorescence Microscopy Imaging

COS-7 cells were stained with BTP (1 µM) for 30 min in Opti-MEM^TM^ reduced serum medium at 37 °C in a 5% CO_2_ humidity-controlled incubator and imaged by using an Olympus FV1000 multiphoton microscope with an oil 25X XLPlanA, 1.05 na, water numerical aperture objective. COS-7 cells stained with BTP were excited in the range 895–995 nm at 10 nm increment intervals by using a tunable Mai Tai^®^ DeepSee^TM^ titanium sapphire laser line (690–1040 nm) and the corresponding emissions were collected from 560 nm to 700 nm. Fluorescence microscopy images were analyzed using the ImageJ (NIH) software for quantification purposes.

### 2.6. Fluorescence Microscopy Imaging of Bacteria

*Bacillus megaterium* was freshly cultured for fluorescence microscopy imaging experiments and stained according to the previously reported methodology [36]. For all imaging experiments, microorganisms were stained with either 5 µM or 10 µM BTP concentrations prepared by diluting 1 µL of the stock solution (1 mM made in DMSO) of the probes into aqueous media. *Bacillus megaterium* was grown in Luria broth (LB) agar plates. A sterilized loop was used to inoculate a single colony of bacteria from a streak plate into microcentrifuge tubes containing the appropriate concentration of the probe in sterile water and samples were incubated for 30 min at room temperature. After staining, a 4 µL volume was spotted on a glass slide and secured using a cover slip and the stained bacteria were then imaged using a fluorescence microscope under 40× and 100× (oil) magnification. Images were acquired by exciting the specimen with the standard Cy3 filter settings (580–620 nm) for emission collection. The bright-field images were acquired for all staining experiments.

### 2.7. Fluorescence Lifetime Studies

Fluorescence lifetime was measured by using a time-correlated single-photon counting (TCSPC) method on an Edinburgh FLS 1000 spectrometer system. The instrument was equipped with a high-speed PMT in cooled housing with a 230–850 nm response. All measurements were performed by exciting the sample (10 µM) solutions using a pulsed diode laser (EPL-510) as the excitation source, providing 506 nm excitation with a pulse duration of 85 ps, while collecting emission data in the 630–640 nm range. All curves were constructed using the OriginPro^®^ 2019 software to generate fluorescence lifetime curves. All lifetime curves were generated in the presence of an instrument reference function (IRF) signal to minimize instrument errors. Spectroscopic grade anhydrous solvents were used for all lifetime analysis experiments. To obtain lifetime data in cellular and physiological environments, a 5 µM final concentration of BTP was used. Four samples containing BTP (5 µM) were prepared in (1) live cells (300,000 cells/mL), (2) FluoroBrite^TM^ DMEM, (3) DPBS, and (4) water. The data acquisition was performed at the observed emission maximum in the steady-state emission spectra.

## 3. Results and Discussion

### 3.1. Photophysical Properties in Solution

**Figure 1:**
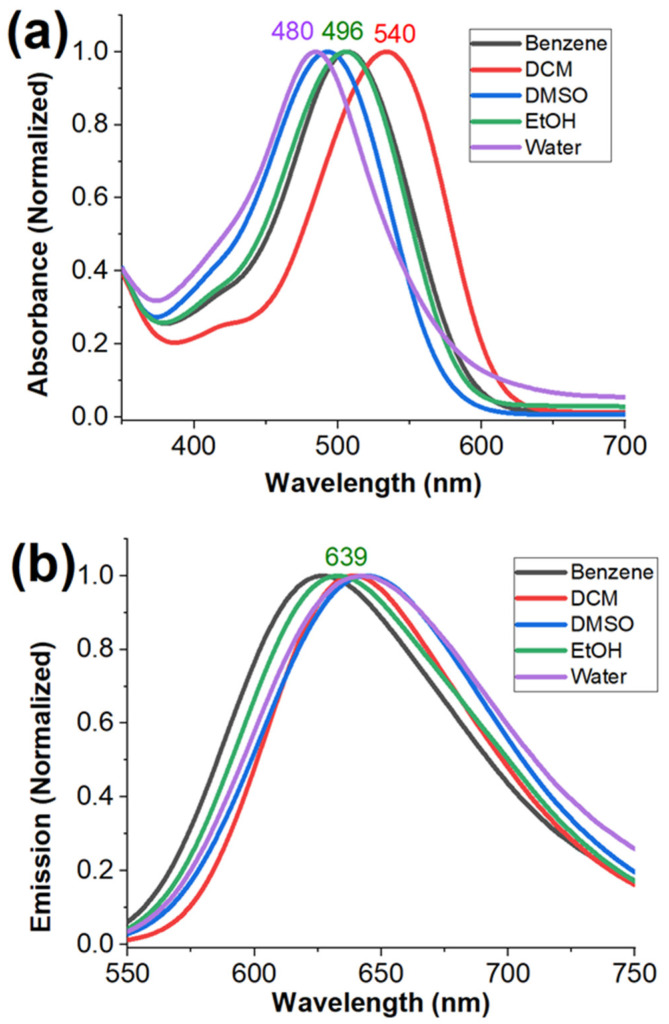
Absorbance (**a**) and emission (**b**) of BTP (1 × 10^−5^ M) in different solvents at room temperature. For the emission spectra (**b**) acquisition, BTP was excited at λ_ex_ ≈ 490 nm, and the emissions were collected from 510 nm to 800 nm.

Steady-state fluorescence and absorbance spectra were acquired for BTP in different solvents, i.e., dichloromethane (DCM), chloroform (CHCl_3_), acetonitrile (ACN), dimethyl sulfoxide (DMSO), dimethylformamide (DMF), ethanol (EtOH), methanol (MeOH), and water, as reported in Table 1 and Figure 1a, b. The acquisition of the optical spectra in benzene and toluene was not successful because of the limited solubility of BTP due to its ionic nature. Solvents were chosen to cover a wide range of polarity, ranging from non-polar (toluene) to moderately polar (DCM and chloroform), polar aprotic (DMSO and DMF), and polar protic solvents (ethanol, methanol, and water). As previously reported, the emission spectra of BTP did not exhibit a noticeable solvatochromic effect with respect to the response to the polarity of the solvent (Figure 1b) [21]. In summary, the emission maximum exhibited a narrow shift (λ_em_ ≈ 638–646 nm) in response to the solvent environments. Apart from DCM and chloroform, BTP only exhibited a slight change in the absorbance maximum (λ_abs_ ≈ 486–498 nm) in response to the solvent polarity (Figure 1a). However, interestingly, BTP exhibited a noticeable bathochromic shift in absorbance spectra in the presence of chlorinated solvents such as DCM (λ_em_ ≈ 540 nm) and chloroform (λ_em_ ≈ 530 nm) (Figure 1b and Table 1). The observed unusual bathochromic shift in the absorbance spectra of BTP can be explained by considering changes in the dielectric environments of the solvent. Previously published work has demonstrated the impact of the solvent’s dielectric nature on the absorbance spectra of a fluorophore [37,38,39,40]. Especially in charged fluorophore systems (i.e., cyanine), solvents with a high dielectric constant (i.e., water) can stabilize the charged species through a solvation effect, which can lower the ground state energy of the fluorophore to result in a hypsochromic shift (i.e., blue-shift) in the absorbance spectra [37,38,39]. On the other hand, solvents with poor dielectric constants (i.e., chloroform, DCM) may destabilize the charged ground state of the fluorophore that can increase the relative ground state energy of the fluorophore [37,38,39]. Therefore, a noticeable bathochromic shift (i.e., red shift) is observed in the absorbance spectra.

The fluorescence lifetime measurements were acquired for BTP (at λ_em_ ≈ 630–640 nm) in different solvents and are reported in Table 1. In summary, the calculated decay curves for the BTP in all organic solvents indicate a single exponential decay in the range of 0.29–0.58 ns (Table 1 and Supplementary Materials Figure S1). The observed single exponential decay suggests that the emission collected at λ_em_ ≈ 640 nm exclusively resulted from the intra-molecular charge transfer state (ICT state). Interestingly, the decay curves in aqueous environments exhibited a two-exponential decay with calculated lifetimes of 0.29 ns (95.65%) and 1.29 ns (4.35%) (Table 1 and Supplementary Materials Figure S1). Unlike many environment-sensitive fluorescent dyes, the fluorescence lifetimes of BTP did not exhibit a traditional solvatochromic response toward solvent polarity (Table 1). This observation is also consistent with the reported steady-state measurements (i.e., absorbance, emission), where BTP was nearly non solvatochromic toward solvent polarity (Table 1). Interestingly, the dielectric constant of the solvent seemed to exhibit a significant impact on the fluorescence lifetime of BTP based on our findings (Table 1 and Supplementary Materials Figure S1). In summary, the fluorescence lifetime of BTP tends to increase in higher dielectric constant solvents, indicating a negative solvatochromic impact toward fluorescence lifetime (Table 1 and Supplementary Materials Figure S1).

### 3.2. Co-Localization and Co-Staining Studies

**Figure 2:**
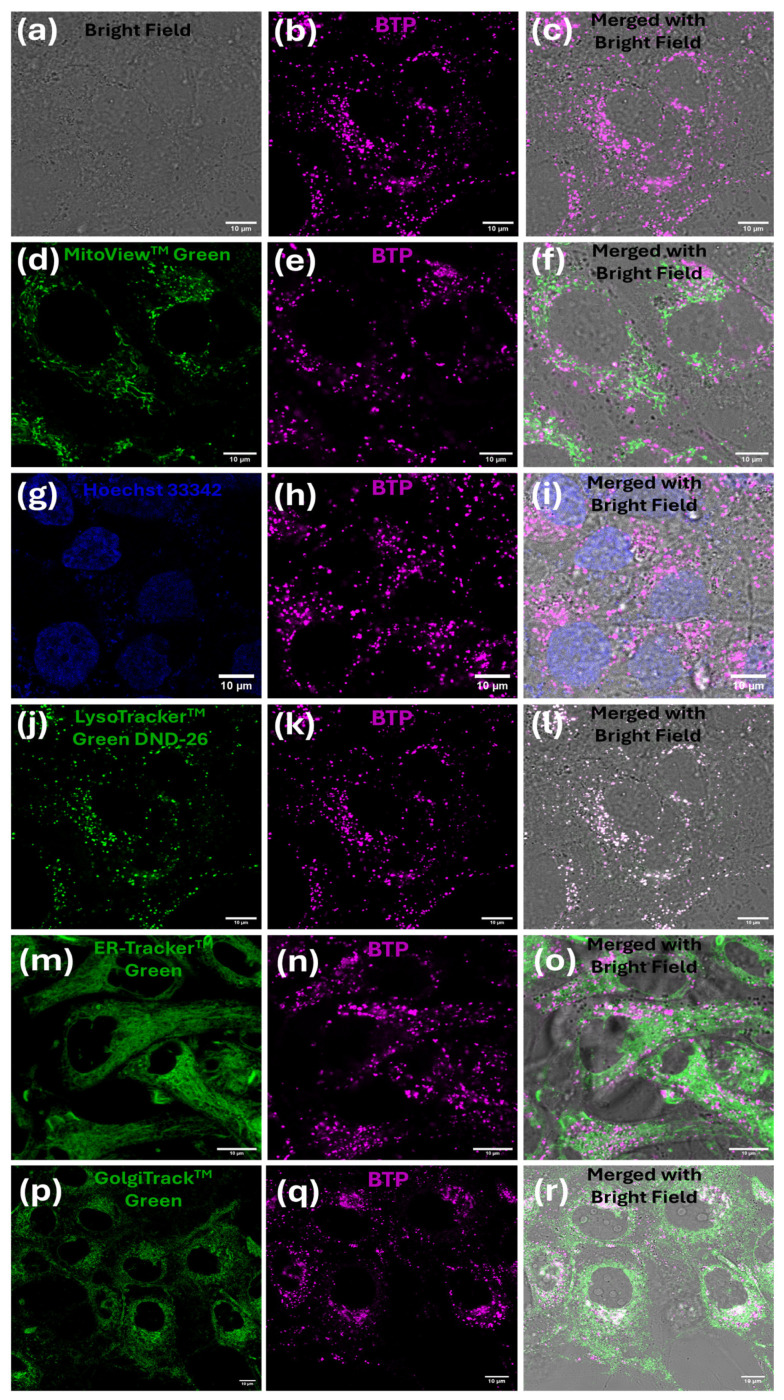
Fluorescence confocal microscopy images of MG-63 cells with BTP (1 µM) in the presence of different organelle markers. Figures (**a**–**l**) represent BTP only (**a**–**c**), with MitoView^TM^ green (**d**–**f**), Hoechst 33342 (**g**–**i**), LysoTracker^TM^ green DND-26 (**j**–**l**), ER-Tracker^TM^ green (**m**–**o**), and GolgiTrack^TM^ green (**p**–**r**). All co-staining experiments were merged with a bright field to show cell boundaries. The staining procedure and the excitation/emission parameters are described in the Methods section. The scale bar represents 10 µm.

**Figure 3:**
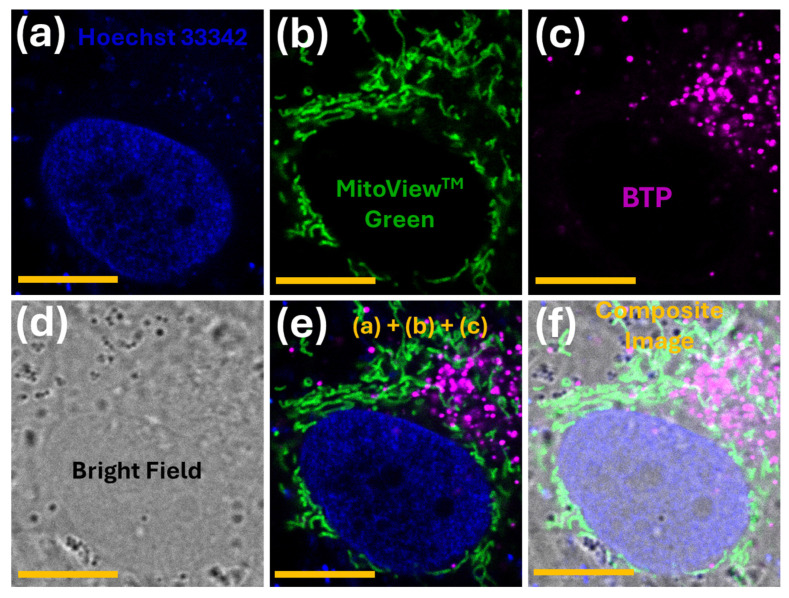
Fluorescence confocal microscopy images of MG-63 cells co-stained with Hoechst 33342 (**a**), MitoView^TM^ green (**b**), and BTP (**c**). Figures (**d**–**f**) represent the bright field (**d**), a merged multichannel image (**e**), and the composite image with the bright field (**f**). The staining procedure and the excitation/emission parameters are described in the Methods section. The scale-bar represents 10 µm.

A systematic co-localization study was performed for BTP (1 µM) with different commercial organelle markers (nucleus, mitochondria, lysosome, endoplasmic reticulum, and Golgi) in MG-63 cells and it is summarized in Figure 2. BTP did not show any acceptable co-localization pattern with these markers, except for commercial lysosome dyes (Figure 2). The observed lysosome specificity and the co-staining compatibility with many different sub-cellular markers clearly indicate the universal applicability of BTP in many bioimaging contexts. In addition, multicolor co-staining experiments were performed for BTP (1 µM) in the presence of different organelle markers (nucleus, mitochondria, and lysosome) to evaluate the probe’s suitability for multicomponent co-staining studies (Figure 3). Based on experimental findings, BTP exhibits a remarkable compatibility for multicomponent co-staining studies, a property which is crucial in modern imaging applications.

### 3.3. Spectral Profiles and Behavior in Intracellular Environments

**Figure 4:**
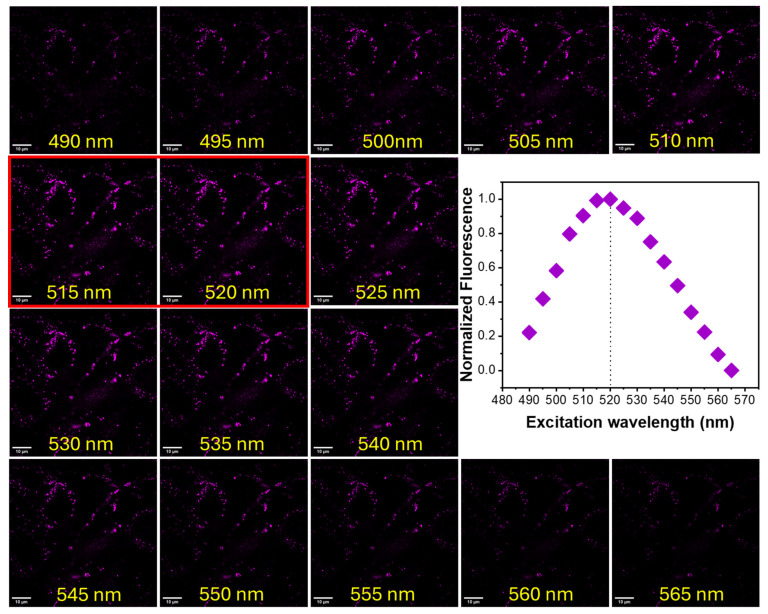
Fluorescence confocal microscopy images of MG-63 cells stained with BTP (1 µM) and excited in the wavelength range 490–565 nm. The emissions were collected from 550 nm to 700 nm.

**Figure 5:**
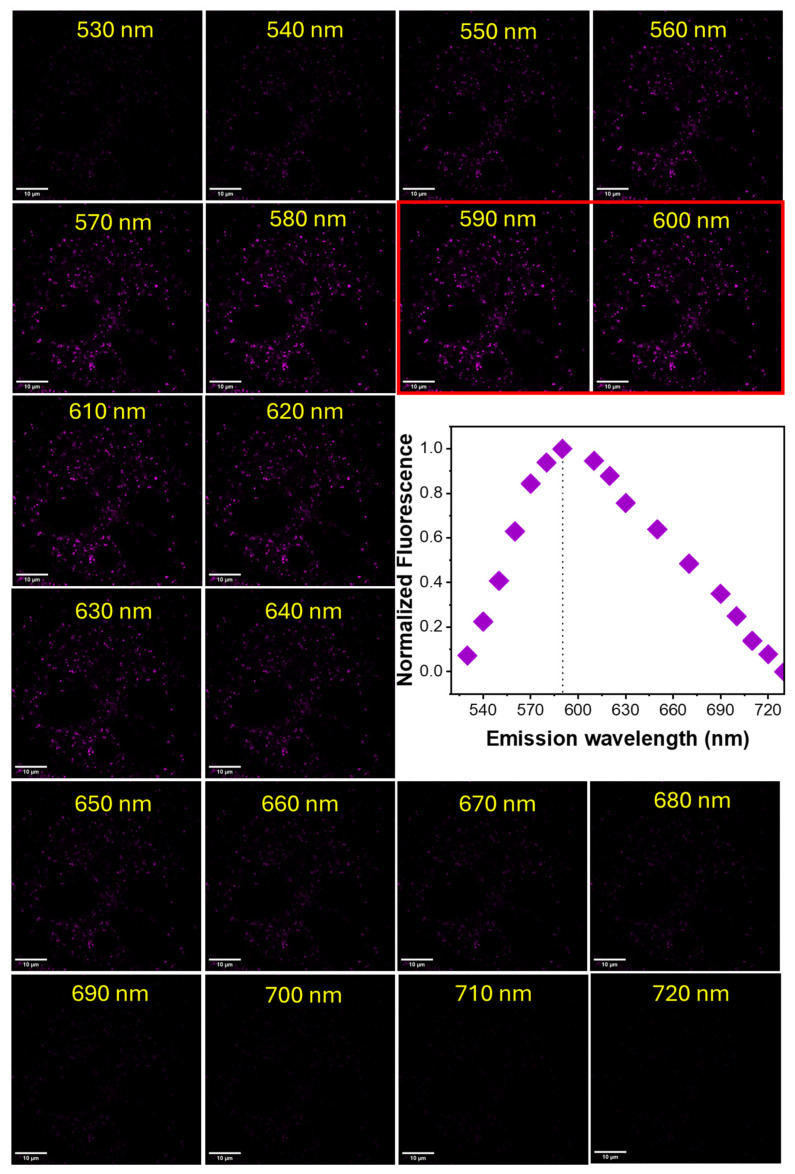
Fluorescence confocal microscopy images of MG-63 cells stained with BTP (1 µM) and excited at a laser wavelength of 520 nm. The emissions were collected from 530 nm to 720 nm at 10 nm intervals.

MG-63 cells stained with BTP (1 µM) were analyzed using a confocal microscope system consisting of a tunable white light laser (WLL). Stained cells were excited within the wavelength range of 490–565 nm at 5 nm intervals (at constant power) and the resulting emission profiles were captured within the 550–700 nm range (Figure 4). The collected fluorescence microscopy images were processed using the ImageJ (Fiji) software to calculate the averaged normalized fluorescence intensity, which was plotted against the excitation wavelength (Figure 4). Based on the computed spectroscopic data, the excitation maximum for BTP in a lysosomal environment was found to be λ_ex_ ≈ 520 nm (Figure 4). The observed excitation maximum in a lysosomal environment was significantly different from the excitation maximum observed in water (λ_ex_ ≈ 480 nm, Table 1) or in aqueous acidic environments (λ_ex_ ≈ 470 nm at pH 4–6) [13]. The steady-state measurements in different solvent environments also indicate a noticeable bathochromic shift in the absorbance spectra in non-polar (i.e., chloroform or dichloromethane) environments (see Table 1). Therefore, the observed bathochromic shift in the excitation profile (compared to aqueous environments) indicates that the localization of the BTP was likely occurring into a relatively hydrophobic lysosomal environment such as a membrane. To further investigate the behavior of the probe in a lysosomal environment, MG-63 cells stained with BTP (1 µM) were excited at 520 nm and the resulting emissions were collected from 530 nm to 720 nm at 10 nm point intervals (Figure 5). The normalized average fluorescence intensities were plotted as a function of emission wavelength (Figure 5). Interestingly, the computed emission maximum was found to occur at λ_em_ ≈ 590 nm (Figure 5). To further validate this unexpected hypsochromic shift in emission spectra, steady-state spectra were recorded for a suspension of MG-63 cells in the presence of BTP (1 µM) (Supplementary Materials Figures S2 and S3). Interestingly, the observed emission maximum was also found to occur at λ_em_ ≈ 590 nm, as observed in the fluorescence microscopy analysis (Supplementary Materials Figures S2 and S3). However, the observed hypsochromic shift in emissions did not occur in aqueous environments in the absence of cells (Supplementary Materials Figure S2). Therefore, based on this spectroscopic evidence, we hypothesize that BTP is likely internalized into/bound to a hydrophobic lysosomal environment (i.e., membrane or a membrane protein), with such binding/internalization-induced geometric changes (i.e., planarity) likely causing the observed hypsochromic shift which is a well-studied phenomenon in similar push–pull-type ICT probes.

**Figure 6:**
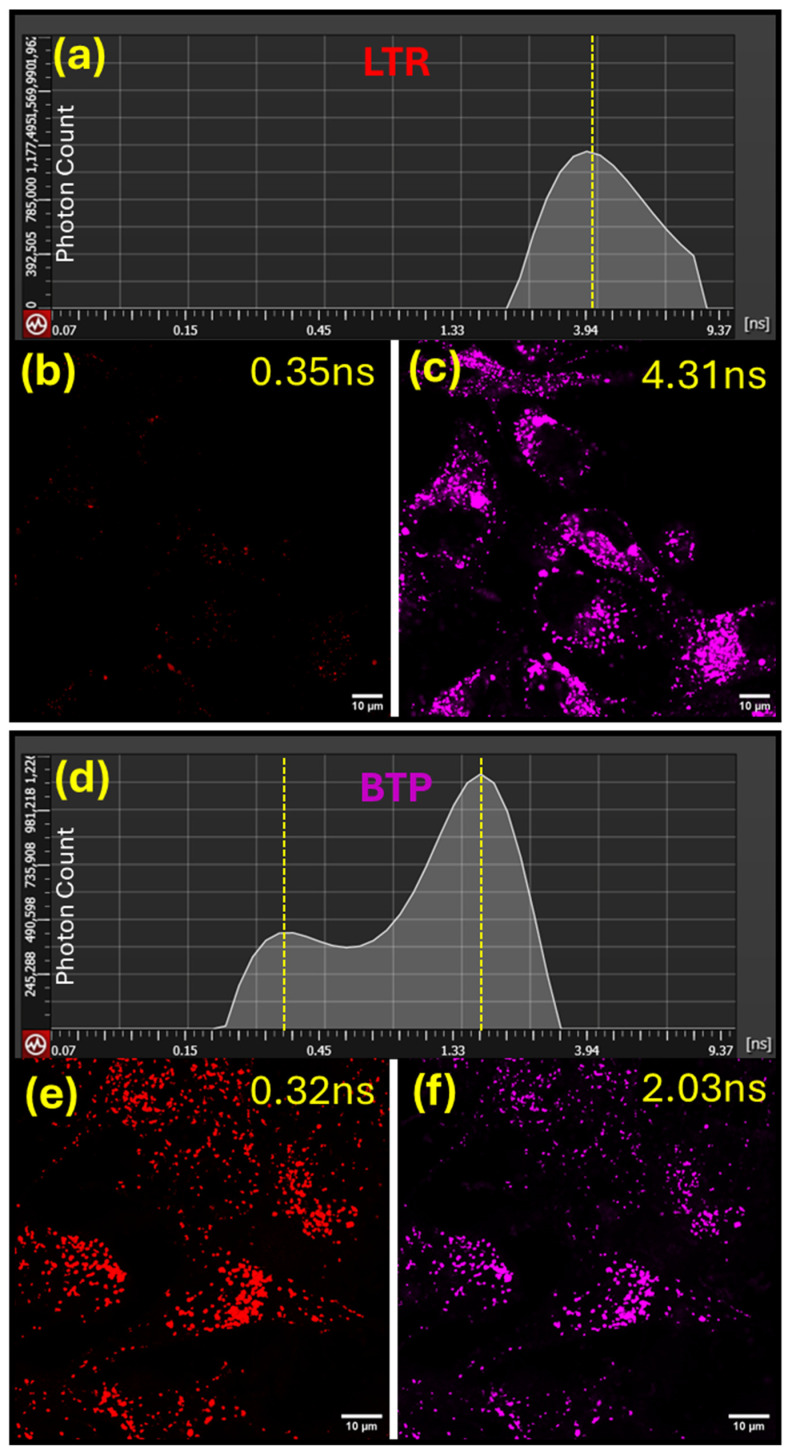
Fluorescence confocal microscopy analysis of MG-63 cells stained with LysoTracker^TM^ red DND-99 (**b**,**c**) and BTP (**e**,**f**) to study their emitted photon population based on the averaged arrival time (AAT). Figures (**a**,**d**) illustrate the emitted photon distribution based on the AAT for LysoTracker^TM^ red DND-99 (**a**) and BTP (**d**).

In order to understand the probe’s microenvironment and spatial distribution changes in cellular lysosomes, MG-63 cells stained with BTP (1 µM) were further analyzed using time-gated emission separation methods, i.e., tau separation, which is based on the fluorescence lifetime of the probe [34,35]. Fluorescence lifetime imaging is a highly sensitive and reliable technique that can accurately determine changes in a fluorophore’s microenvironment (i.e., polarity, viscosity, etc.) [4,34,41]. The fluorescence microscopy imaging performed, based on the average photon arrival time, revealed two distinguishable red emissions (λ_em_ ≈ 590 nm) resulting from lysosomes stained with BTP in the MG-63 cells (Figure 6). The relatively shorter emission component, with an average photon arrival time of ͳ_1_ = 0.32 ns, exhibited a lower abundance (25%), whereas the emission component with a longer average photon arrival time ͳ_2_ = 2.03 ns exhibited a higher abundance (75%) based on time-gated photon counting experiments performed during the fluorescence microscopy imaging (Figure 6). One could argue that the observed two populations of red-emitting photons may likely occur due to the dynamic distribution of BTP between hydrophobic (i.e., membrane) and hydrophilic (i.e., lumen) lysosomal environments (BTP_(membrane)_ <-> BTP_(lumen)_)_._ However, according to the steady-state measurements (see Table 1), BTP is nearly non-fluorescent in aqueous environments (Φ_fl_ < 0.005). Therefore, the possibility of a bright red emission from the lysosomal lumen is not favorable. Another possible working model that can explain the two different emission populations observed entails considering the distribution of BTP in lysosomal membrane regions while interacting with a macromolecular target (i.e., membrane protein) that would lead to two populations (i.e., BTP_(bound)_ <-> BTP_(unbound)_). However, further structure-based studies are required to confirm potential macromolecular targets for BTP in lysosomal environments. As a control experiment, MG-63 cells were stained with commercial LysoTracker^TM^ red DND-99 and the resulting photon populations were analyzed (Figure 6). In sharp contrast to BTP-stained cells, LysoTracker^TM^-red-stained cells exhibited a single population of photons with an average arrival time of ͳ = 4.31 ns (Figure 6). Commercial LysoTracker^TM^ dyes have been designed to accumulate in the acidic lysosomal lumen, with the observed single population of photons strongly supporting this working model [42,43]. The consistency of the observed dual emissive states for BTP lysosomal environments were further confirmed by performing time-resolved microscopy imaging in several other cell lines, including HeLa, A172, HepG2, and U251 (Supplementary Materials Figure S4).

### 3.4. Time-Lapse Imaging

**Figure 7:**
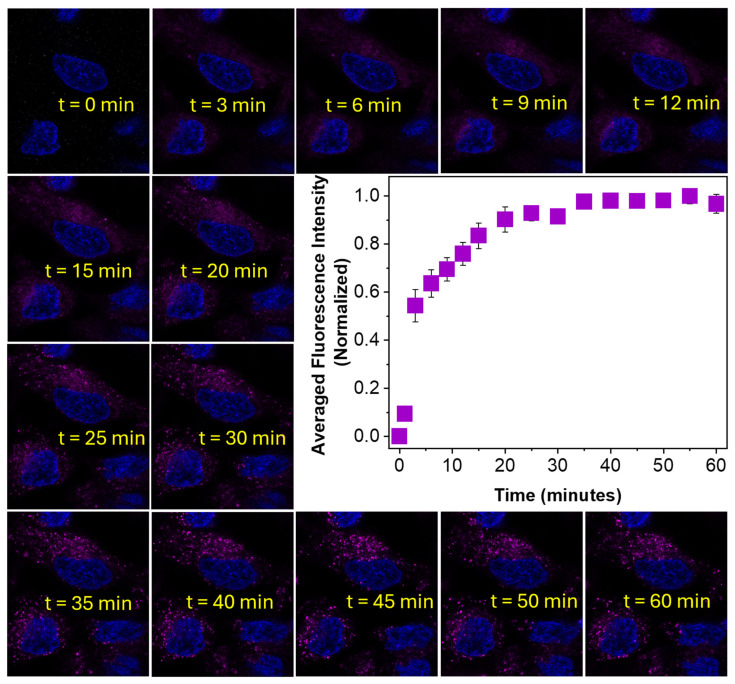
Time-lapse fluorescence confocal microscopy images of MG-63 cells stained with BTP (1 µM). Cells were pre-stained with Hoechst 33342 and BTP was added at the time point t = 0 min. Cells were sequentially excited at 520 nm and the emissions were collected from 550 nm to 700 nm.

A detailed internalization study was performed for BTP in MG-63 cells to visualize its internalization in real-time via time-lapse fluorescence confocal microscopy (Figure 7). Thus, BTP (1 µM) was introduced into MG-63 cells pre-stained with Hoechst 33342 and fluorescence microscopy images were obtained at 3–5 min time intervals (Figure 7). The fluorescence microscopy images acquired were analyzed and the average normalized fluorescence intensity was plotted as a function of time (Figure 7). The acquired data clearly indicates that BTP was internalized into MG-63 cells within a 30 min period, with a noticeable increase in the fluorescence emission observed during this period, reaching stabilization after 30 min (Figure 7). The stable emission signal after 30 min further confirms the completion of the internalization of BTP into lysosomal environments (Figure 7). The observed data also well aligned with our previously proposed internalization mechanism, where BTP molecules are transported into live cells via endosomal vesicles (i.e., early endosomes (EE)) which are then transformed (i.e., endosomal maturation) into cellular lysosomes over time (~30 min). Considering the membrane retaining the ability of the probe, the latter is likely to be localized in the membrane regions of the mature lysosomes. Since this dynamic EE-to-lysosome maturation process takes about ~30 min, the obtained time-lapse imaging provides strong visual evidence in support of the endosome-to-lysosome transformation. It is also important to notice that no noticeable background interference was produced by the MG-63 cells stained with BTP during the time-lapse imaging, a phenomenon which also provides strong evidence in support of the universal application of this probe skeleton as a “wash-free” lysosome imaging dye.

### 3.5. Bacteria Imaging Ability

**Figure 8:**
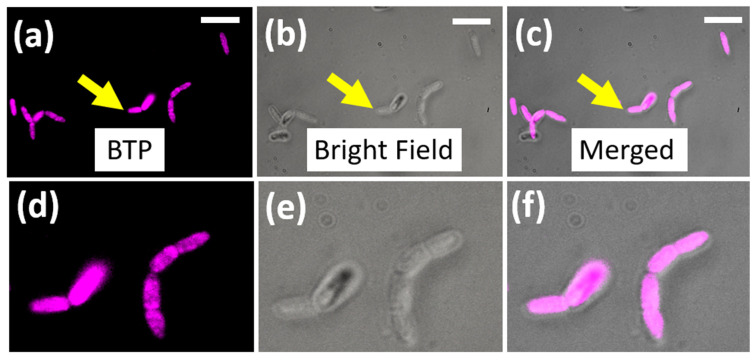
(**a**–**f**) Fluorescence microscopy images of *Bacillus megaterium* cells stained with BTP (5 µM) for 30 min. Images were acquired by exciting the stained bacterial cells with standard Cy3 filter settings (580–620 nm) for the emission collection. The scale bar indicates 5 microns.

In order to investigate the microorganism (i.e., bacteria) visualization ability of BTP, we cultured *Bacillus megaterium* in growth media and stained it with different concentrations of BTP (5–10 µM), as described in the Methods section. Interestingly, the microorganisms stained with BTP exhibited excellent bright emissions with relatively low probe concentrations, such as 5 µM, thus indicating the potential application of BTP as a staining agent for bacteria (Figure 8). In addition, the observed uniform staining pattern suggests that the probe is likely distributed throughout the membrane regions of the bacteria (Figure 8) [36,44]. The observed intense fluorescence signal with no detectable background indicates the superior performance of BTP as a wash-free staining agent for bacteria.

### 3.6. Two-Photon Excitability and Applications

**Scheme 2:**
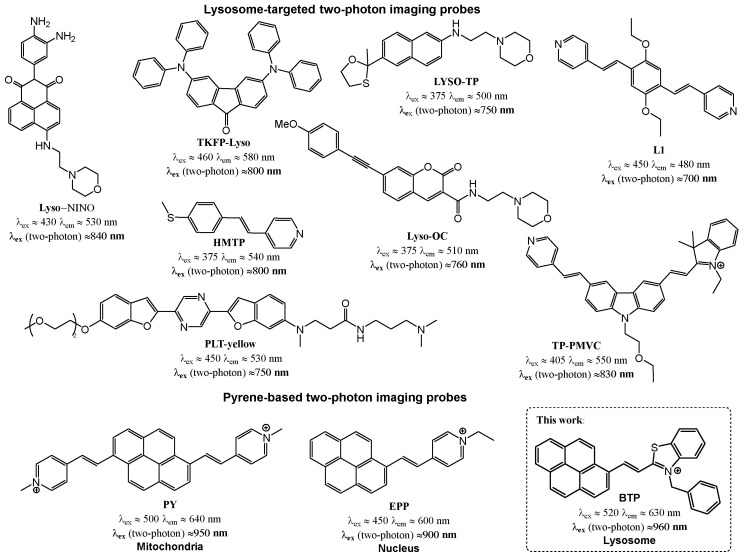
A representative summary showing the chemical structures of different lysosomes targeting two-photon probes developed within the past decades (**top**) and a summary of pyrene-based two-photon excitable probes developed to date for cell imaging applications (**bottom**) [27,36,46,47,48,49,50,51,52,53].

**Figure 9:**
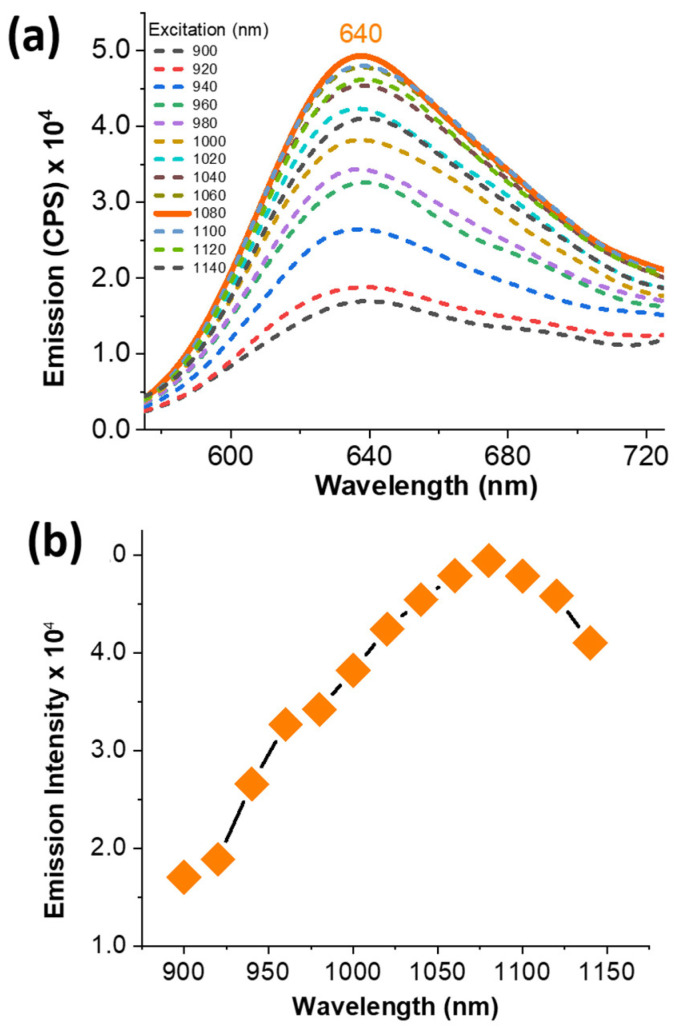
The two-photon excitation spectra of BTP (10 µM) in ethanol at room temperature (**a**) and the emission intensity of the two-photon fluorescence as a function of excitation wavelength (**b**). The emission spectra were collected from 580 nm to 720 nm and the excitations were performed from 900 nm to 1140 nm.

**Figure 10:**
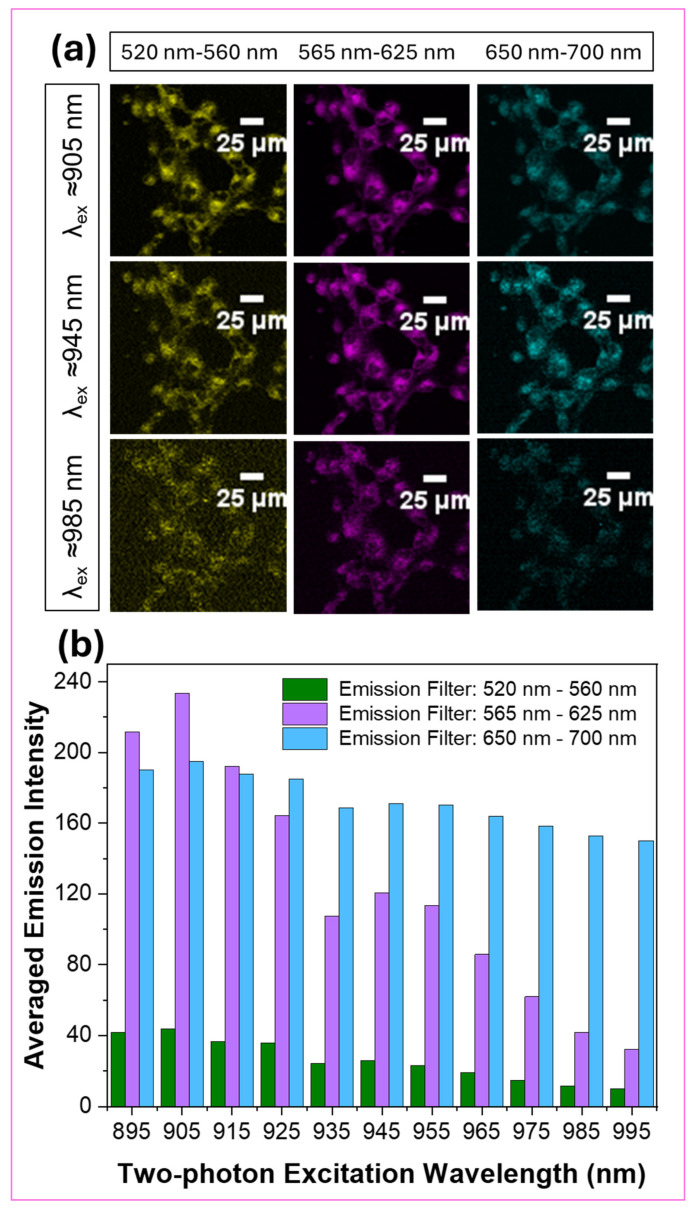
(**a**,**b**) Two-photon fluorescence microscopy images obtained for the COS-7 cells stained with BTP (1 µM) while being excited in the 895–995 nm wavelength range. The emissions were collected from 520 nm to 700 nm.

Fluorescent imaging dyes containing rigid polycyclic aromatic skeletons are well-known for their characteristic two-photon excitability (Scheme 2) [27,45]. In addition, several pyrene-based imaging dyes have been described because of their two-photon excitability, as indicated in Scheme 2. In order to investigate the probe’s two-photon excitability, steady-state emission spectra were acquired for BTP in ethanol while exciting it in the 900–1140 nm wavelength range (Figure 9). The acquired emission spectra exhibited a noticeable two-photon excitability for BTP, with the excitation maximum found at λ_TP_ ≈ 1075 nm (Figure 9). Inspired by this outcome, we performed two-photon fluorescence microscopy imaging on COS-7 cells stained with BTP (1 µM) (Figure 10). Stained cell samples were excited within the wavelength range of 895–995 nm at 10 nm intervals and the resulted emissions were collected using four different emission filters at 460–500 nm (filter cube 1), 520–560 nm (filter cube 2), 565–625 nm (filter cube 3), and 650–700 nm (filter cube 4) (Figure 10 and Supplementary Materials Figures S5–S7). Interestingly, BTP produced a bright red emission at low staining concentrations, such as 1 µM, indicating an outstanding performance as a promising two-photon excitable dye (Figure 10). As observed in the single-photon fluorescence confocal microscopy imaging, BTP exhibited the highest emission intensity within the 565–625 nm (filter cube 3) wavelength range and exhibited no noticeable emissions within the filter cube 1 settings (i.e., 460–500 nm) (Figure 10 and Supplementary Materials Figures S5–S7). The two-photon excitation maximum was observed at λ_ex_ ≈ 905 nm (Figure 10 and Supplementary Materials Figures S5–S7).

## 4. Conclusions

In summary, detailed photophysical properties and imaging applications are evaluated for a red-emitting pyrene-based π-acceptor dye (BTP). BTP exhibits a bright red emission and a large Stokes’ shift Δλ > 100 nm owing to its rigid co-planar geometry that allows for strong ICT interactions. The fluorescence lifetime-based analysis concludes that the ICT-driven red emission λ_em_ ≈ 640 nm is the dominating radiative species at the excited state. BTP exhibits an excellent co-localization ability which can be used with a wide range of fluorescent organelle markers without interference. The excitation spectra analysis for BTP in intracellular environments indicates no significant changes in the spectral profile, suggesting that no structural changes take place during the cellular localization. The observed significant blue shift in the emission spectra (λ_em_ ≈ 690) in the cellular environments affords convincing evidence in support of co-localization/stabilization into a hydrophobic cellular environment (i.e., membrane). Time-lapse fluorescence microscopy analysis confirms the wash-free application of BTP as a potential candidate for real-time imaging owing to its ultra-low background interference. Time-resolved fluorescence microscopy imaging studies confirm the possible existence of BTP in two different microenvironments within cellular lysosomes. BTP also exhibits an excellent ability to stain bacterial species such as *Bacillus megaterium*, indicating its universal application potential as an imaging dye. Finally, BTP also exhibits potential to be utilized as an imaging dye for two-photon microscopy imaging with an excitation maximum at λ_TP_ ≈ 905 nm. Therefore, red-emitting push–pull probes such as BTP can be useful during biomedical imaging applications owing to their excellent photophysical performance and high compatibility with various imaging applications.

## Figures and Tables

**Table 1:** Spectroscopic properties of BTP.

Solvent	DCM	CHCl_3_	ACN	DMSO	DMF	EtOH	MeOH	Water
**λ_abs_ (nm)**	540	530	495	490	491	498	496	480
**λ_em_ (nm)**	638	640	643	645	646	639	642	644
**∆λ (nm)**	98	110	153	155	155	143	144	164
**ϕ_fl_**	0.33	0.29	0.33	0.68	0.40	0.24	0.28	<0.005
ꜫ **(M^−1^cm^−1^)**	37,023	32,456	31,482	36,470	35,700	32,428	29,647	23,162
**τ (ns)**	0.35	0.27	0.34	0.58	0.51	0.36	0.29	0.29 (95.65%) 1.28 (4.35%)

## Data Availability

Data is contained within this manuscript and Supplementary Materials and no additional data is available.

## Supplementary Materials

The following supporting information can be downloaded at: https://www.mdpi.com/article/10.3390/bios14120612/s1, Figure S1: Fluorescence lifetime decays obtained for BTP (10 µM) in different solvents; Figure S2: Emission spectra obtained for LysoTracker^TM^ red DND-99 (1 µM) and BTP (1 µM) in a culture medium (DMEM) at room temperature; Figure S3: Fluorescence lifetime data obtained for LysoTracker^TM^ red DND-99 (1 µM) and BTP (1 µM) in a culture medium (DMEM); Figure S4: Fluorescence confocal microscopy data obtained for the emitted photon populations of LysoTracker^TM^ red DND-99 (LTR) and BTP in different cell lines based on their averaged arrival time (AAT); Figures S5–S7: Two-photon fluorescence microscopy images obtained for COS-7 cells stained with BTP; Figure S8: Characterization data for BTP.
