# Supplementary material for: Exploring Imaging Applications of a Red-Emitting π-Acceptor (π-A) Pyrene-Benzothiazolium Dye
Source: Biosensors (Basel). 2024 Dec 13;14(12):612. doi: 10.3390/bios14120612 (PMC11674487; doi:10.3390/bios14120612)
Supplement: Supplementary file 1 [file biosensors-14-00612-s001.zip › biosensors-3319168-supplementary.pdf]

Exploring Imaging Applications of a Red-Emitting π-Acceptor (π-A) Pyrene-benzothiazolium Dye

Chathura S. Abeywickrama[1*], Enya Huang[1], Wenhui Yan[1], Michael A. Vrionides[2], Paaramitha Warushavithana[3], Kristen A. Johnson[4], Robert V. Stahelin[5], Yi Pang[6], Tomoyasu Mani[2], and Kaveesha J. Wijesinghe[3]

Supporting Information

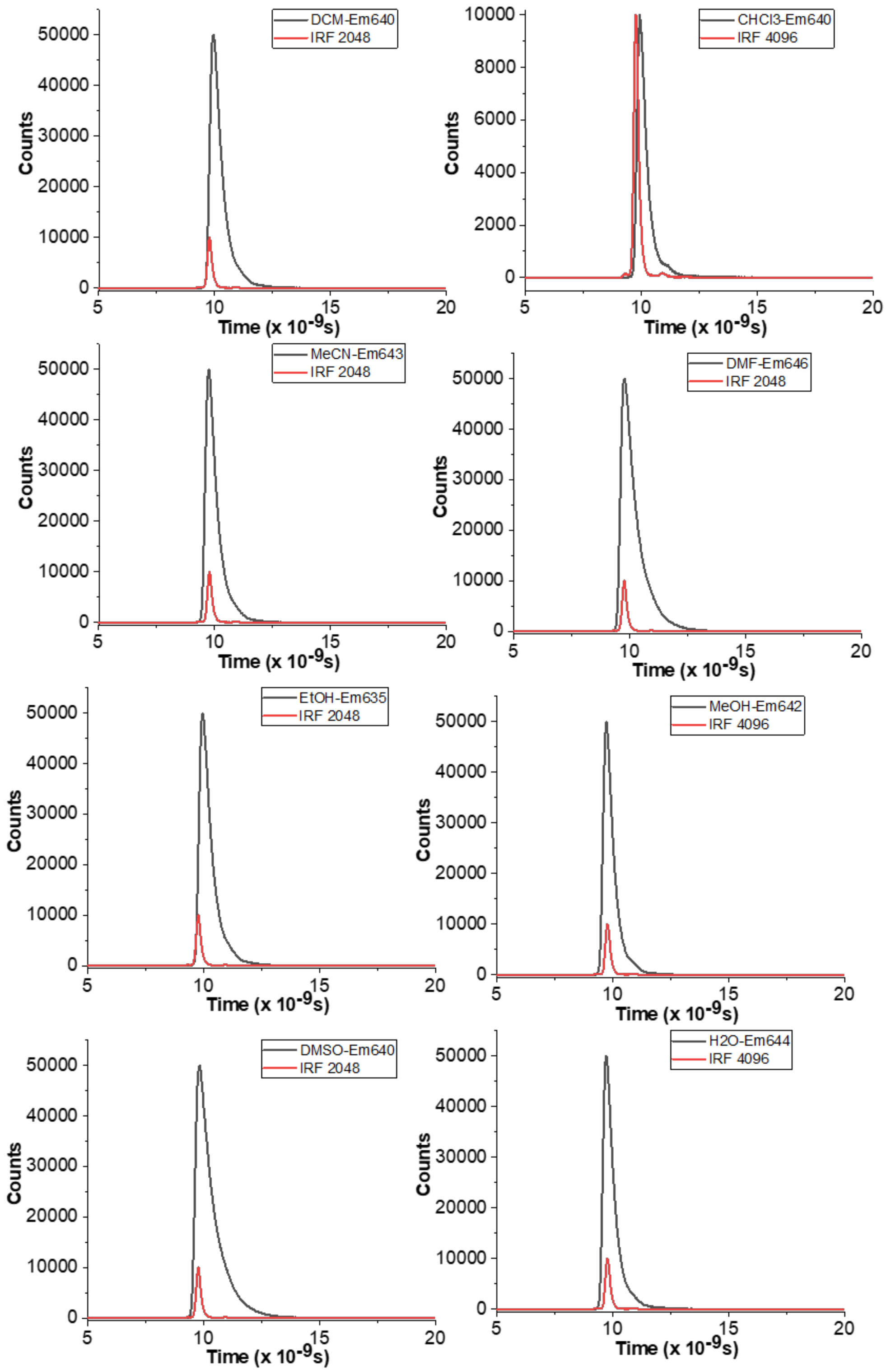

Figure S1. Fluorescence lifetime decays obtain for **BTP** (10 μM) in different solvents at room temperature. The corresponding emission collection wavelengths are shown in the figures. Samples were excited by a 506 nm pulse laser.

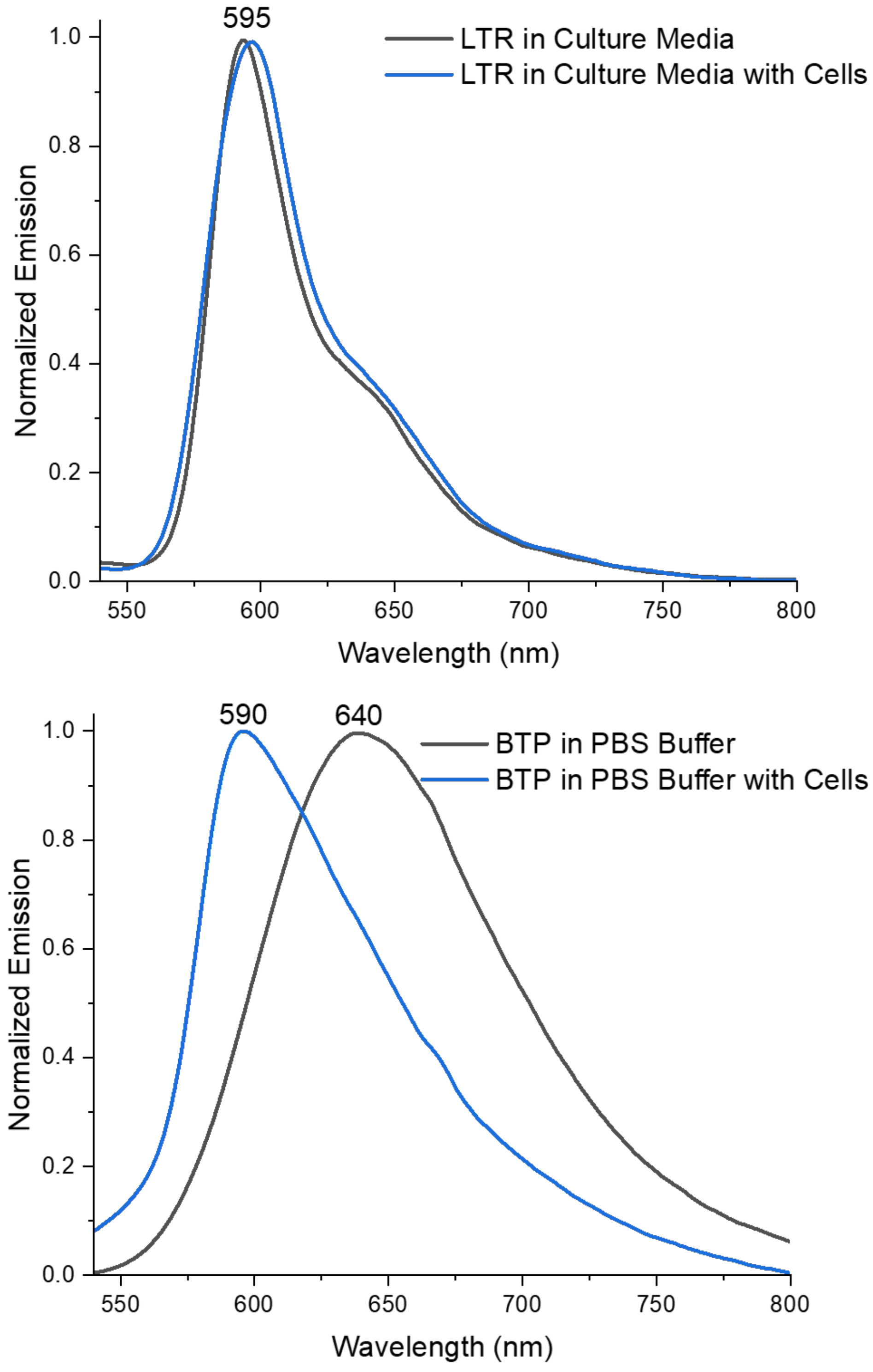

Figure S2. Emission spectra obtained for LysoTracker™ Red DND-99 (1 μM): top and **BTP** (1 μM): bottom in culture media (DMEM) at room temperature in the absence and presence of MG-63 cells in suspension. Both probes were excited at 530 nm to collect the emission spectra.

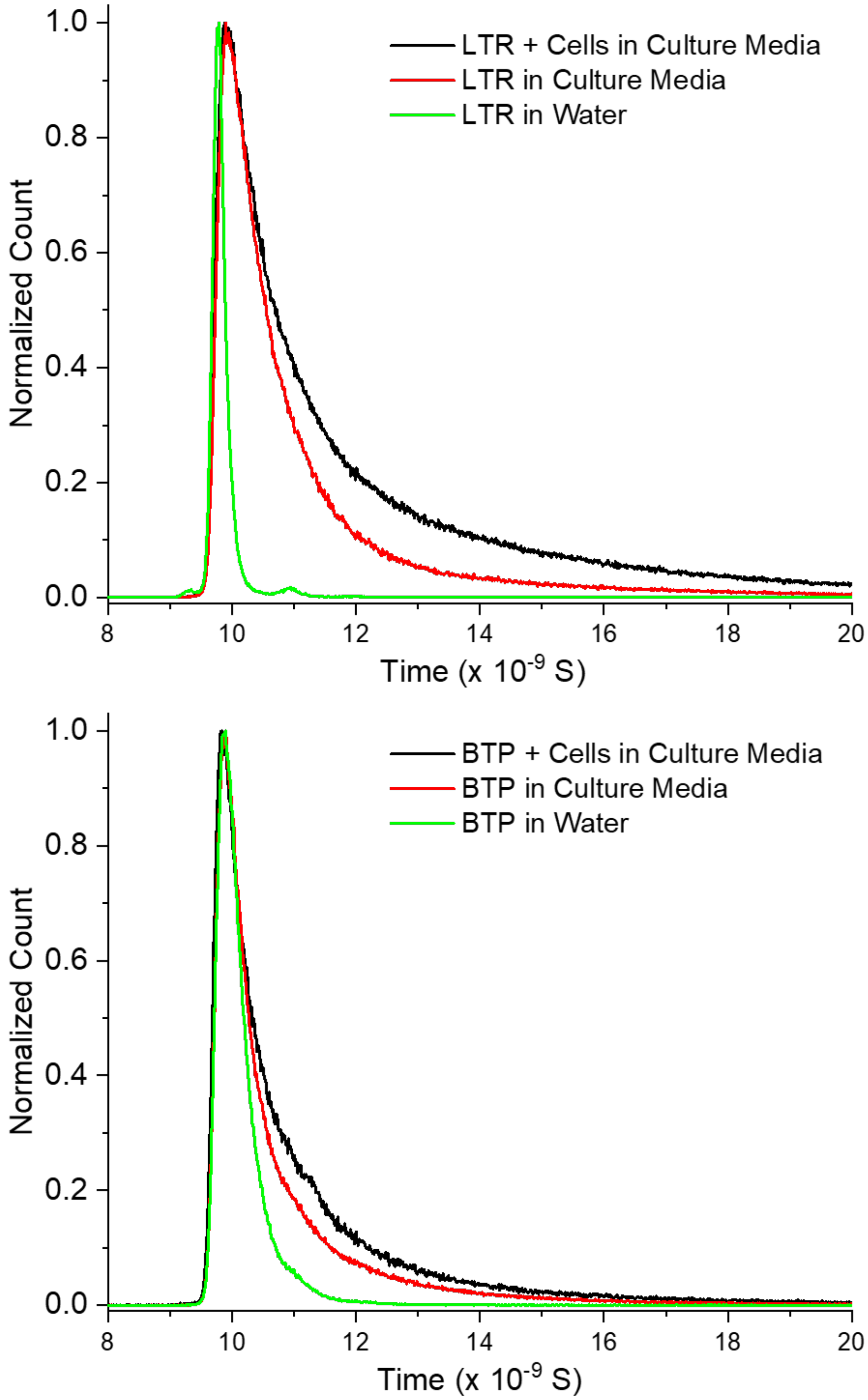

Figure S3. Fluorescence lifetime data obtained for LysoTracker™ Red DND-99 (1 μM): top and **BTP** (1 μM): bottom in culture media (DMEM) at room temperature in the absence and presence of MG-63 cells in suspension. Samples were excited by a 506 nm pulse laser.

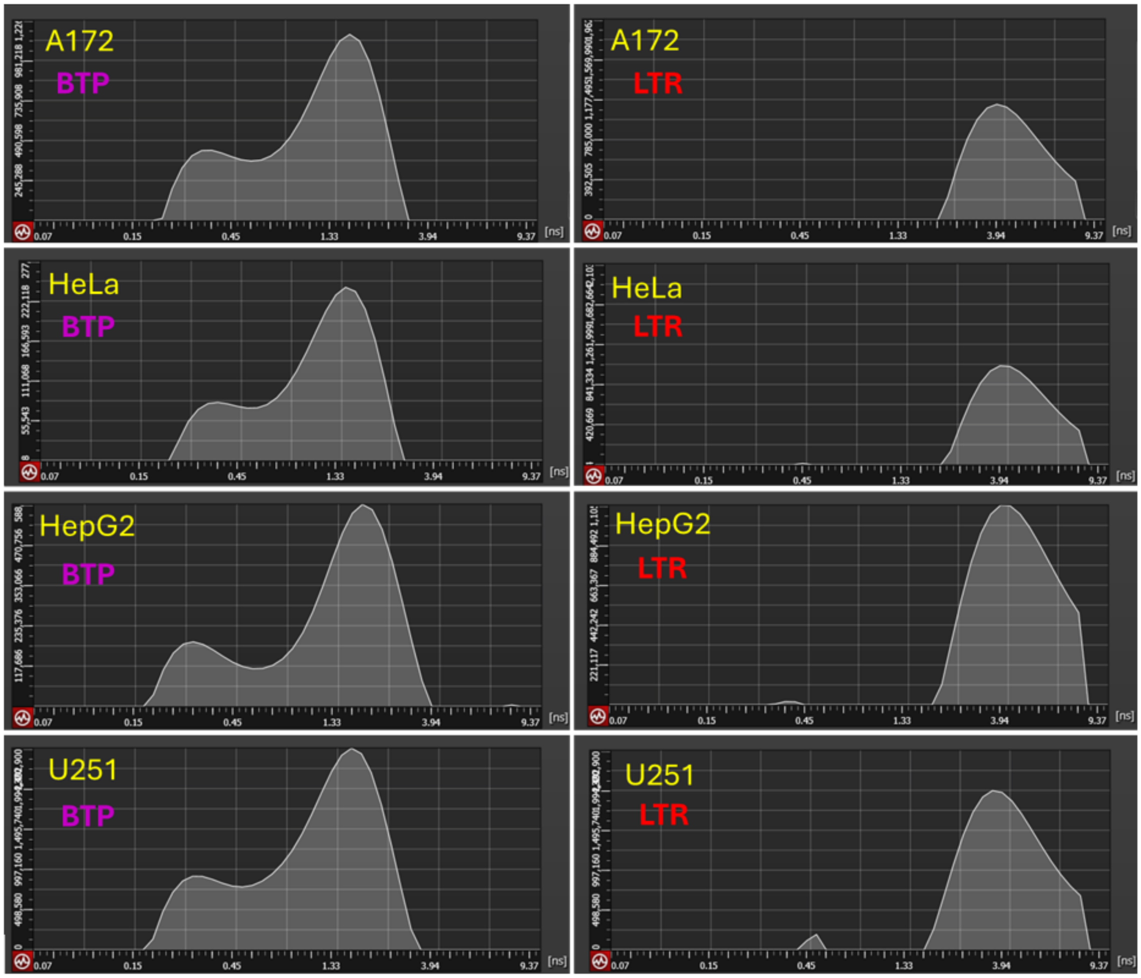

Cell Line	τ1 (ns)	τ2 (ns)
**BTP**		
A172	0.33	2.03
HeLa	0.37	1.70
U251	0.35	2.02
HepG2	0.35	2.31

Figure S4. Fluorescence confocal microscopy data obtained for the emitted photon populations of LysoTracker™ Red DND-99 (LTR) and **BTP** in different cell lines based on to their based on their averaged arrival time (AAT).

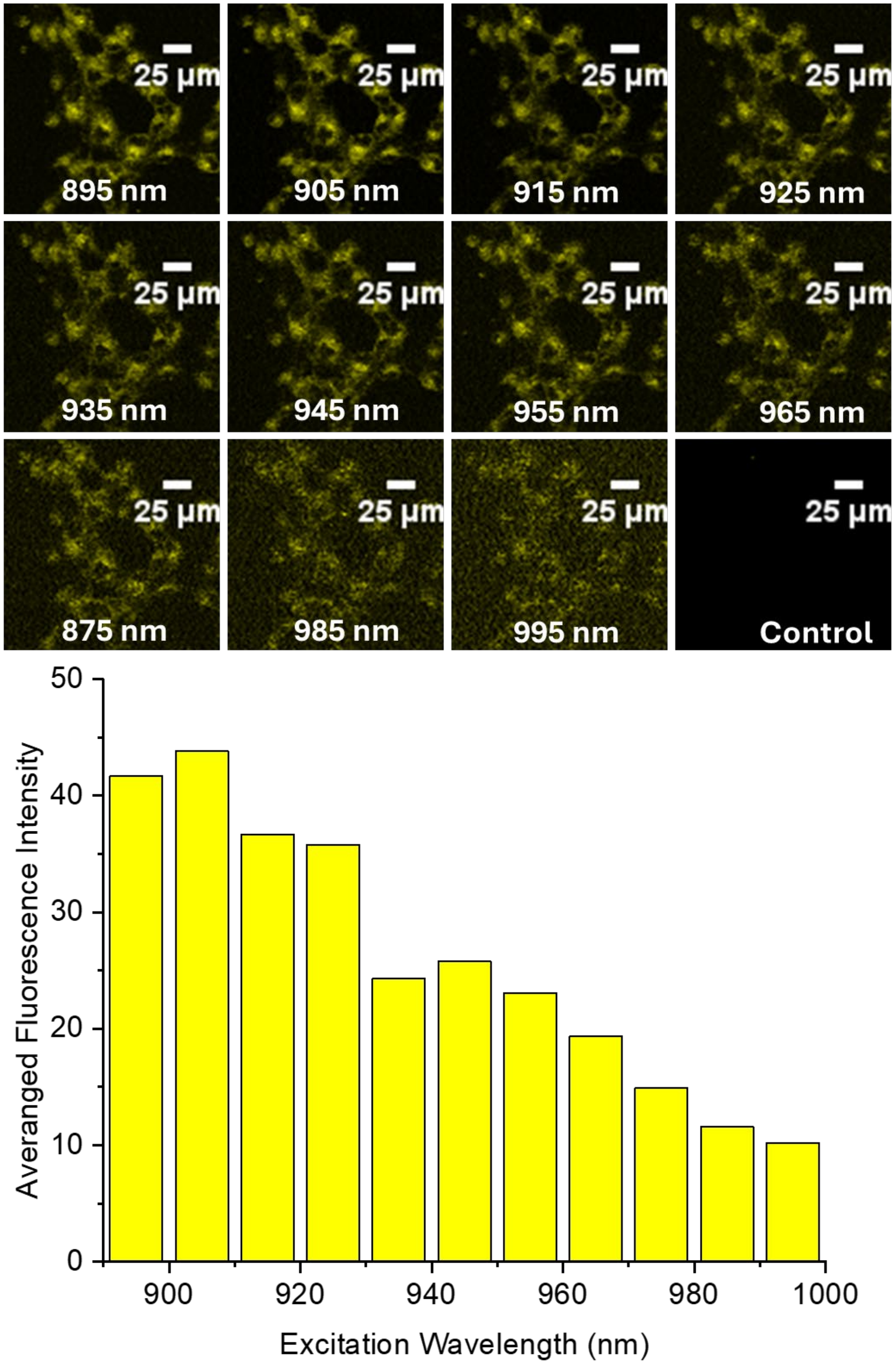

Figure S5. Two-photon fluorescence microscopy images obtained for COS-7 cells stained with **BTP** (1 μM) while exciting from 895 nm to 995 nm wavelength range. The emissions were collected from 520 nm to 560 nm (filter cube 2).

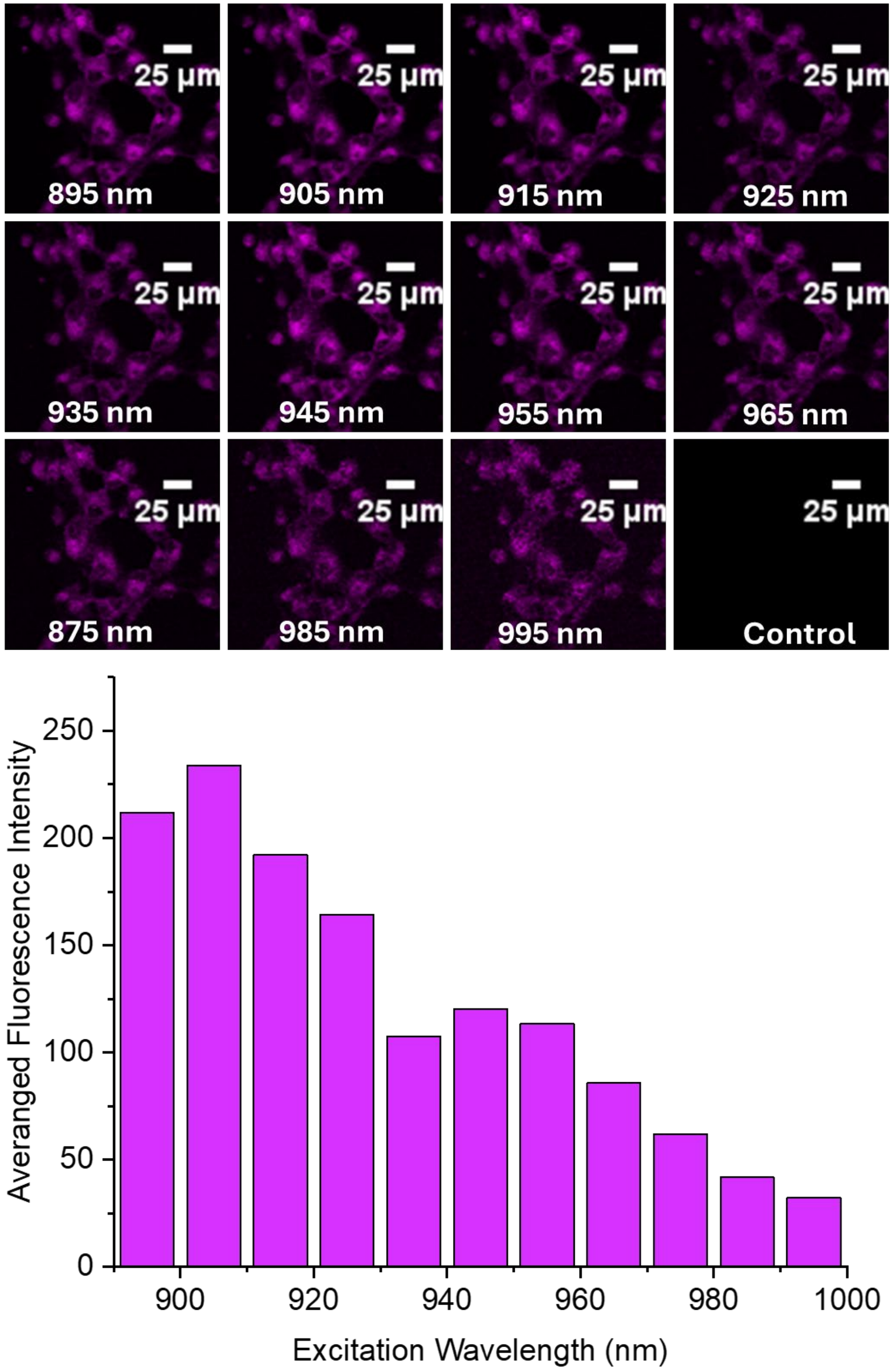

Figure S6. Two-photon fluorescence microscopy images obtained for COS-7 cells stained with **BTP** (1 μM) while exciting from 895 nm to 995 nm wavelength range. The emissions were collected from 565 nm to 625 nm (filter cube 3).

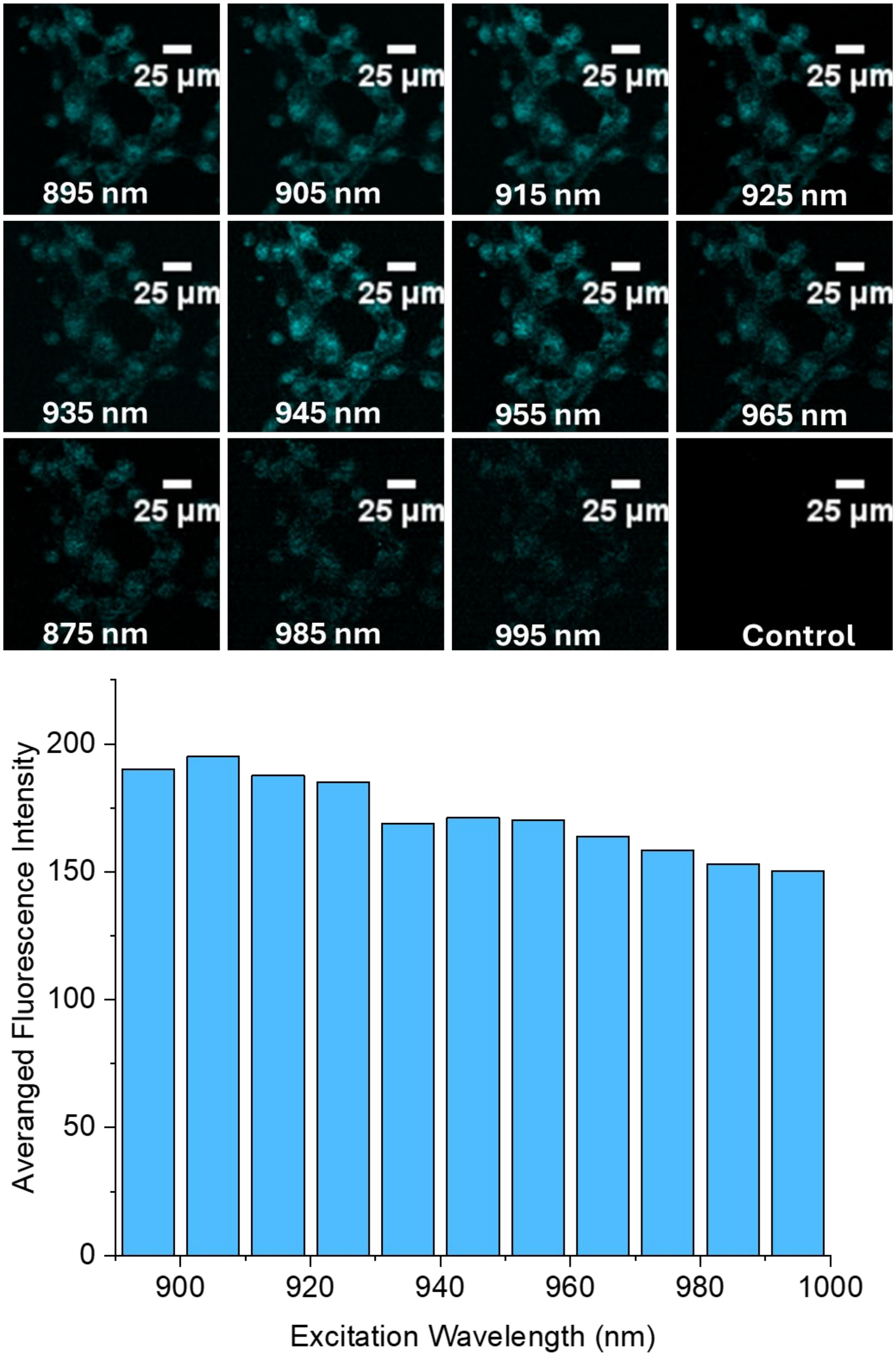

Figure S7. Two-photon fluorescence microscopy images obtained for COS-7 cells stained with **BTP** (1 µM) while exciting from 895 nm to 995 nm wavelength range. The emissions were collected from 650 nm to 700 nm (filter cube 4).

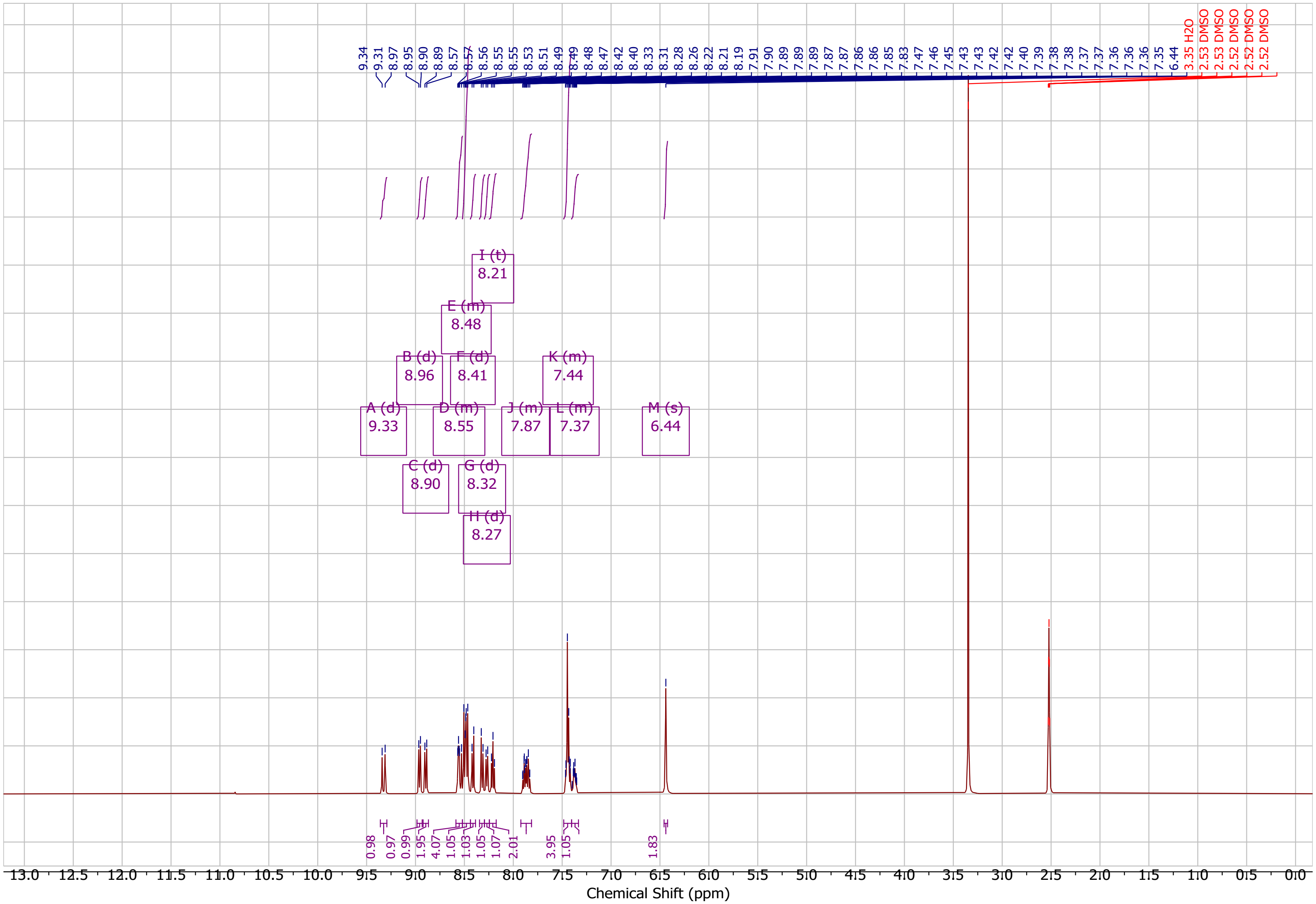

Figure S8.1 ^{1}HNMR spectra acquired for **BTP** in DMSO-*d6*.

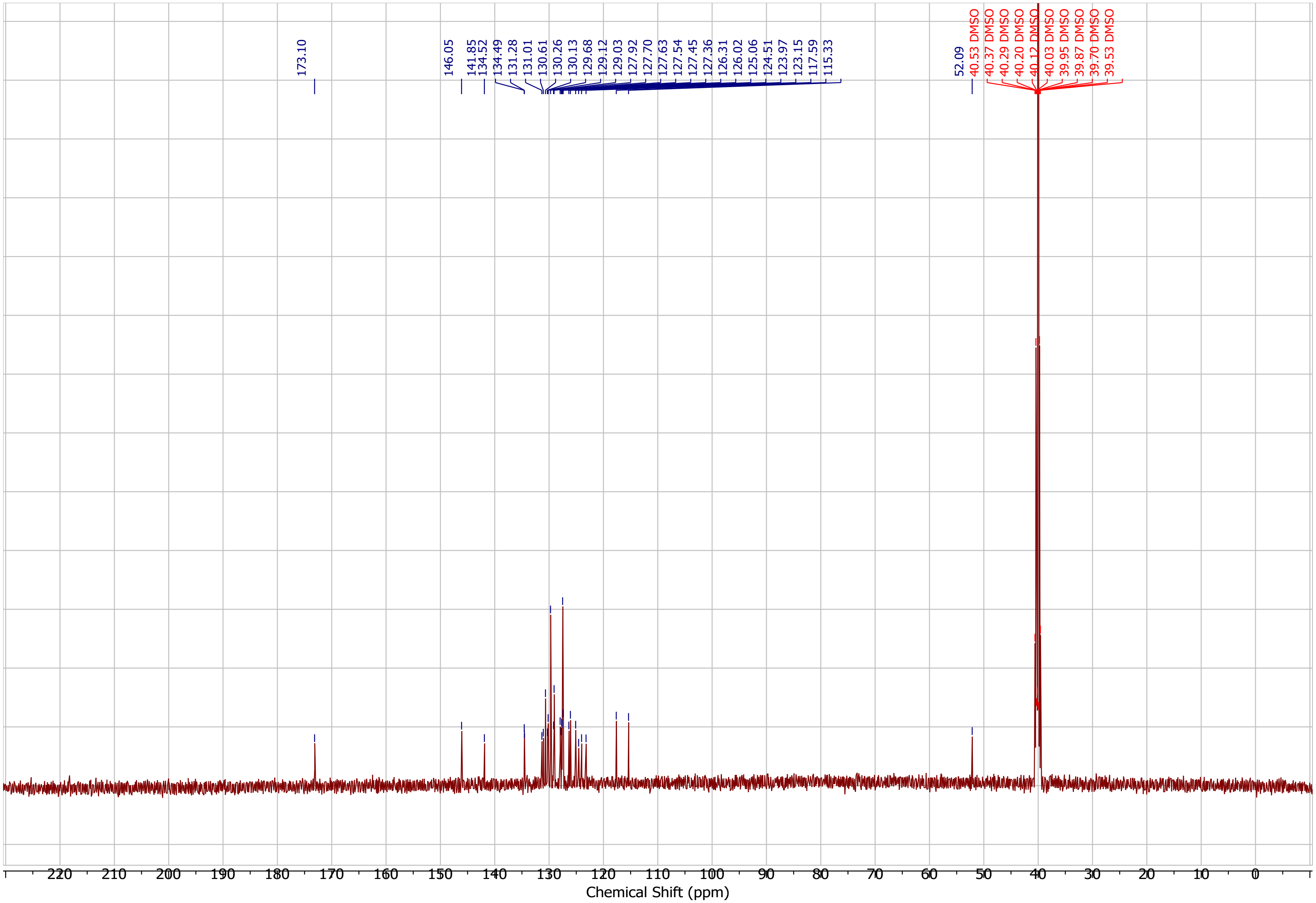

Figure S8.2 ^{13}CNMR spectra acquired for **BTP** in DMSO-*d6*.

13C NMR (126 MHz, DMSO) δ 173.10, 146.05, 141.85, 134.52, 134.49, 131.28, 131.01, 130.61, 130.26, 130.13, 129.68, 129.12, 129.03, 127.92, 127.70, 127.63, 127.54, 127.45, 127.36, 126.31, 126.02, 125.06, 124.51, 123.97, 123.15, 117.59, 115.33, 52.09, 40.53, 40.37, 40.29, 40.20, 40.12, 40.03, 39.95, 39.87, 39.70, 39.53.

^{1}H NMR (500 MHz, DMSO) δ 9.33 (d, *J* = 15.4 Hz, 1H), 8.96 (d, *J* = 8.4 Hz, 1H), 8.90 (d, *J* = 9.4 Hz, 1H), 8.59 – 8.52 (m, 2H), 8.52 – 8.43 (m, 4H), 8.41 (d, *J* = 8.9 Hz, 1H), 8.32 (d, *J* = 8.8 Hz, 1H), 8.27 (d, *J* = 8.2 Hz, 1H), 8.21 (t, *J* = 7.6 Hz, 1H), 7.92 – 7.81 (m, 2H), 7.48 – 7.38 (m, 4H), 7.40 – 7.33 (m, 1H), 6.44 (s, 2H).

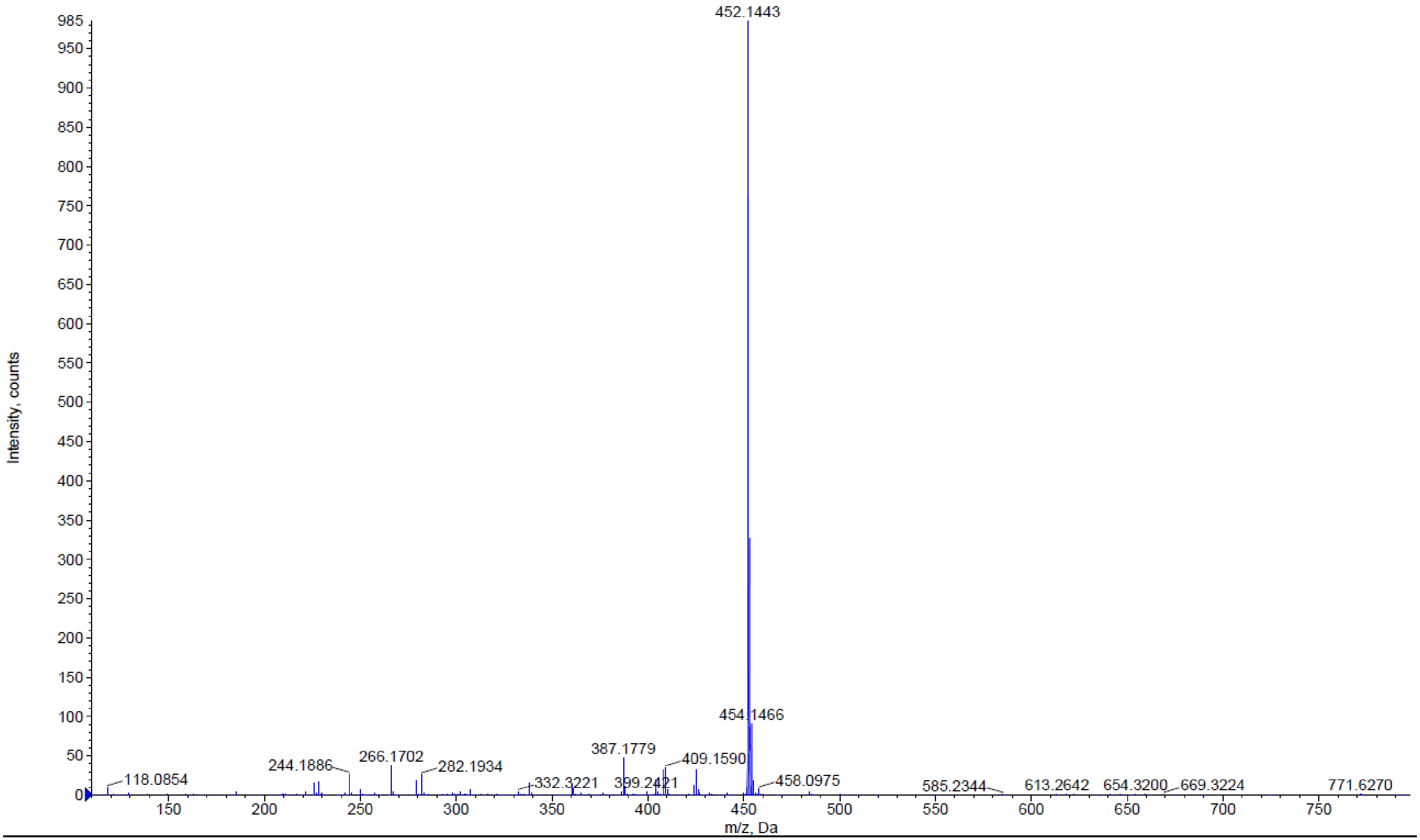

Figure S8.3 HRMS spectra acquired for **BTP** {[$C_{32}H_{22}NS$]$^+$}.

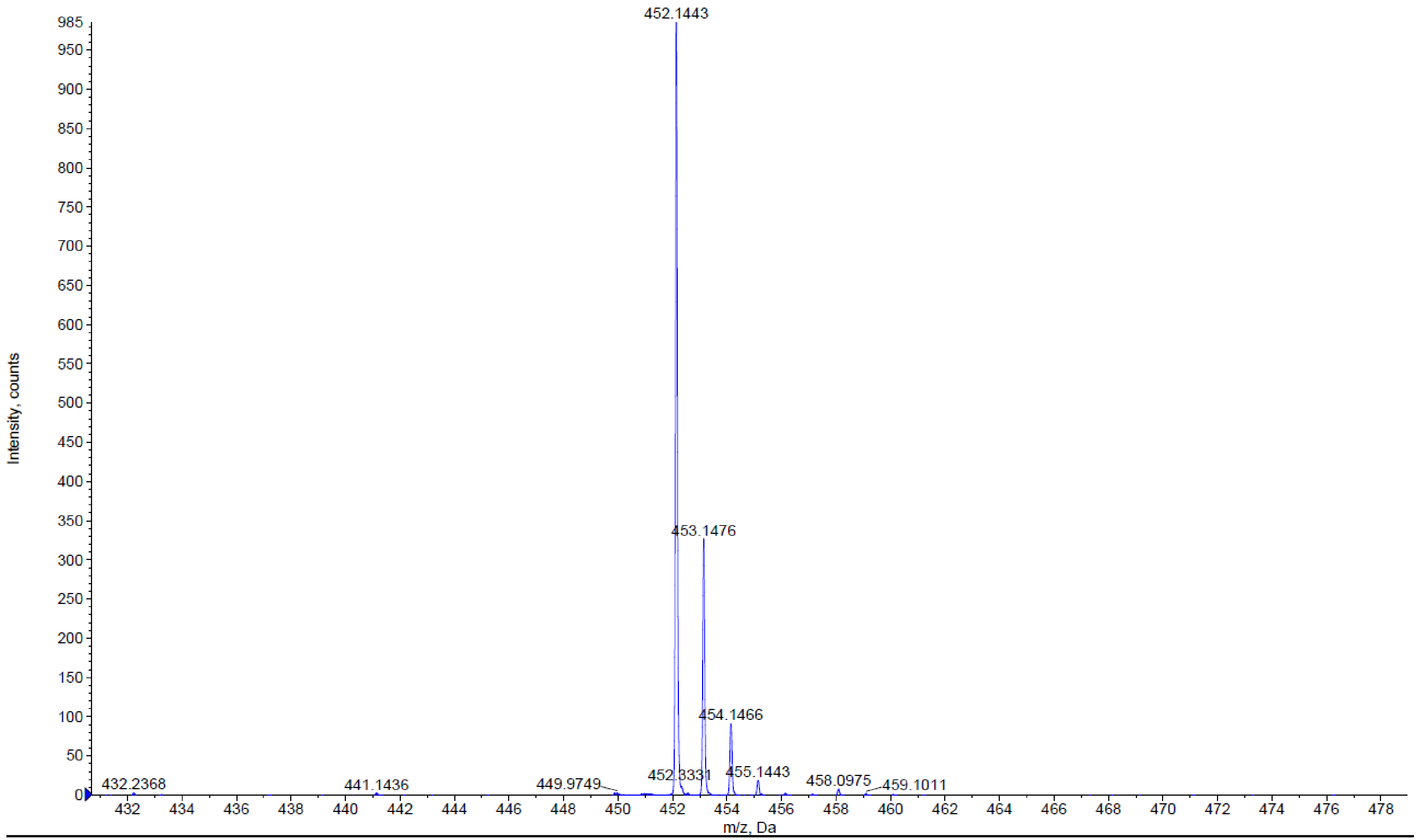

Figure S8.4 HRMS spectra (isotopic pattern) acquired for **BTP** {$C_{32}H_{22}NS]^{+}$}.

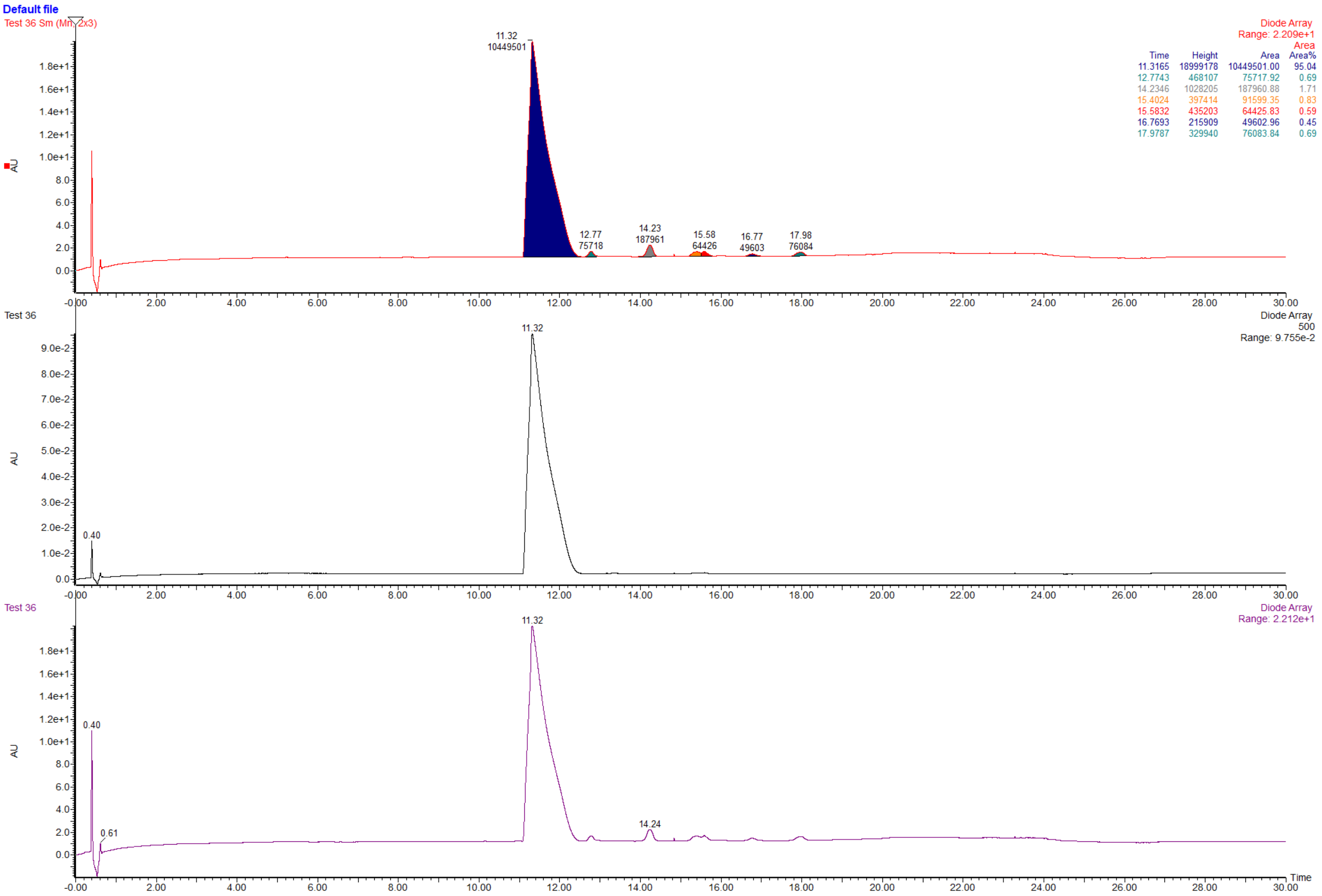

Time	Height	Area	Area%
11.3165	18999178	10449501.00	95.04
12.7743	468107	75717.92	0.69
14.2346	1028205	187960.88	1.71
15.4024	397414	91599.35	0.83
15.5832	435203	64425.83	0.59
16.7693	215909	49602.96	0.45
17.9787	329940	76083.84	0.69

Figure S8.5 HPLC trace analysis for **BTP** in Water (A)/Acetonitrile (B) (0.1 % v/v Formic acid) mobile Phases. The gradient was run from 5% B to 95 %B over a period of 30 minutes. The peak purity of **BTP** is ~ 95%.
